# Trends in NBA and Euroleague basketball: Analysis and comparison of statistical data from 2000 to 2017

**DOI:** 10.1371/journal.pone.0223524

**Published:** 2019-10-07

**Authors:** Radivoj Mandić, Saša Jakovljević, Frane Erčulj, Erik Štrumbelj

**Affiliations:** 1 University of Belgrade, Faculty of Sport and Physical Education, Belgrade, Serbia; 2 Faculty of Sports, University of Ljubljana, Ljubljana, Slovenia; 3 Faculty of Computer and Information Science, University of Ljubljana, Ljubljana, Slovenia; Texas A&M University, UNITED STATES

## Abstract

We analyse and compare NBA and Euroleague basketball through box-score statistics in the period from 2000 to 2017. Overall, the quantitative differences between the NBA and Euroleague have decreased and are still decreasing. Differences are even smaller after we adjust for game length and when playoff NBA basketball is considered instead of regular season basketball. The differences in factors that contribute to success are also very small—(Oliver’s) four factors derived from box-score statistics explain most of the variability in team success even if the coefficients are determined for both competitions simultaneously instead of each competition separately. The largest difference is game pace—in the NBA there are more possessions per game. The number of blocks, the defensive rebounding rate and the number of free throws per foul committed are also higher in the NBA, while the number of fouls committed is lower. Most of the differences that persist can be reasonably explained by the contrasts between the better athleticism of NBA players and more emphasis on tactical aspects of basketball in the Euroleague.

## Introduction

Basketball experts are unanimous that the the National Basketball Association (NBA) league features most of the best players in the world and is where the highest level of basketball is played. However, most experts are also of the opinion that the differences between NBA teams and top European teams, most of which play in the Euroleague, arguably the second-best competition, are decreasing.

Games between NBA and European teams are few and far between, for promotional purposes and lacking competitiveness. Therefore, a direct comparison of teams’ or players’ performance and quality across competitions is difficult, in most cases based on expert opinion and often speculative. Comparisons are additionally complicated by non-negligible differences in rules, the most important being game duration, three-point shot arc distance, and three second violation in defense in the NBA.

However, the 21st century has brought major changes to European basketball. First, game rules, regulated by the International Basketball Federation (FIBA), have changed substantially and in the direction of making them more similar to NBA rules. These changes include, since the 2000/2001 season, 4 periods of 10 minutes (4 periods of 12 minutes in NBA), 24-second shot-clock, 8-seconds advancing the ball over the center line, and, since the 2010/2011 season, moving the three-point arc further away, to 6.75 m from the basket (7.24 meters in NBA), the way of charging unsportsmanlike foul, changes to how and when the 24-second shot-clock is reset and modifying the shape of the key from a trapezoid to a rectangle. Second, the biggest teams in Europe created a new competition—the Euroleague. It was assumed that these rule changes and the Euroleague competition would have an effect on how basketball is played in Europe and gradually decrease the differences in game-related indicators between NBA and Euroleague.

Over the years, numerous methods of registration and analysis have been created to measure game variables; from simple stats sheets completed by assistant coaches to the fully computerized procedures that record all the significant game-performance indicators [1, 2]. Today, a standard set of statistics (also known as box-score statistics or just box-score) is recorded for almost all professional basketball games. The box-score is a useful tool for coaches during the game, for preparing for the next game, analysing past games, and evaluating the performance of a team or a player at the end of the competition period.

Previous studies based on game-related statistics have shown that differences between winning and losing teams are mostly due to defensive rebounds, 2-point field-goal percentages and assists [1, 3, 4, 5, 6, 7, 8, 9, 10, 11, 12, 13, 14]. Current research indicates the discriminative game-related statistics of team performances vary according to several contextual factors, such as game location (home and away), game type (regular season and playoff) and game final score differences (close, balanced and unbalanced games). Several studies have also been dedicated to home-team advantage, which is always present but varies from competition to competition [14, 15, 16, 17, 18, 19, 20]. In this study we focus on the highest level of competition.

Studies that analyse trends in basketball in high-level competitions based on game-related indicators are scarce. According to [21] moving the shot clock from 30 to 24 seconds resulted in a greater number of possessions per game and a higher number of points per game in the first ten seasons of Euroleague after the change was made (2000/2001—2010/2011). Moving the 3-point arc resulted in a lower frequency and a lower percentage of the three-point shots and an increased number of two-point shots, however, with a slightly decrease in shooting percentage. Those changes affected other game-related indicators in the Euroleague, such as a greater number of total rebounds, fewer personal fouls and free-throws. They speculated that in future seasons three-point shot percentages might increase in European basketball as a result of players adapting and modifications to the training process. A study of the fundamental structural features of basketball offense in NBA and the Euroleague [22] shows many similarities, in particular, equal pace and game dynamics.

One of the few studies that compared NBA and European basketball [23] revealed that NBA teams use the overhead pass while European teams use the bounce pass to pass the ball to the player near the basket. Unlike European teams, more players were found in post up positions in the NBA, not just centers. As a consequence, there is more inside game in NBA than in European basketball. An analysis of shot technique [24] concluded that two discernible differences between NBA and European basketball are that dunks are more frequent and hook shots are less frequent in the NBA compared to European basketball, which can be attributed to better athleticism of NBA players. The effect of situational variables (shot location, transition/set, etc.) on shot types and shot success were found to be very similar.

In this paper we investigate the trends in NBA and Euroleague basketball in the period from 2000 to 2017 using box-score statistics. As far as the authors are aware, [21] is the only study that analyses the Euroleague over a longer period of time and the are no similar studies for the NBA or comparisons of the two competitions. We also explore how much of the variability in team success can be explained retrospectively.

In the remainder of the paper we first describe the data and methods used, followed by the results with discussion and conclusions.

## Methods

### Data

Using custom R [25] scripts we gathered box-score data for all NBA and Euroleague games for all seasons in the period 2000/01—2016/17 from

- www.basketball-reference.com and
- www.euroleague.net.

In total, there were 41050 regular season and 2764 playoff games in the NBA and 5032 regular season and 2484 top 16 and playoff games in the Euroleague. The raw dataset with all the statistics used in this paper is available as supplementary material S1 Data.

We use these acronyms for the basic box-score count statistic totals:

**Tot.FTA** free-throws attempted
**Tot.FTM** free-throws made
**Tot.P2A** two-point shots attempted
**Tot.P2M** two-point shots made
**Tot.P3A** three-point shots attempted
**Tot.P3M** three-point shots made
**Tot.AST** assists
**Tot.BLK** blocks
**Tot.STL** steals
**Tot.TOV** turnovers
**Tot.FCM** fouls committed
**Tot.FRV** fouls received
**Tot.ORB** offensive rebounds
**Tot.DRB** defensive rebounds
**Tot.TRB** all rebounds

We use the derived factor $\text{F}.\text{NP}=\text{P2A}+\text{P3A}+\frac{1}{2}\text{FTA}+\text{TOV}$ as a proxy for number of possessions per game.

With the prefix **Paceadj**, we are referring to the pace-adjusted total for that game—the total divided by the number of possessions NP. In the analyses we focus on these pace-adjusted total counts and not the total counts. This adjustment is necessary when comparing count statistics across competitions with different game length (and possibly different pace) as is the case for NBA (12 minute quarters) and Euroleague (10 minute quarters). If such an adjustment is not made then the 8 minute difference (20% of Euroleague game time) would by itself result in significant differences for all count statistics. By adjusting for the number of possessions, we can analyse differences that are just due to differences in game length or pace.

We also use other derived factors (denoted by prefix **F**). Note that we omit the Tot prefix in these definitions, but all the terms on the right-hand sides refer to total counts:

**F.FTpct** free-throw shooting percentage $=\frac{\text{FTM}}{\text{FTA}}$
**F.P2pct** two-point shooting percentage $=\frac{\text{P2M}}{\text{P2A}}$
**F.P3pct** three-point shooting percentage $=\frac{\text{P3M}}{\text{P3A}}$
**F.FTperF** free throws per foul committed $=\frac{\text{FTA}}{\text{FRV}}$
**F.ORB** offensive rebounding rate $=\frac{\text{O}}{\text{O}\;+\;\text{opponents}\;\text{D}}$
**F.EFG** effective field goal percentage $=\frac{\text{P2M}+1.5\text{P3M}}{\text{P2A}\;+\;\text{P3A}}$
**F.TOR** turnover rate $=\frac{\text{TOV}}{\text{NP}}$
**F.FTR** free throw rate $=\frac{\text{FTM}}{\text{P2A}\;+\;\text{P3A}}$
**F.DRB** defensive rebounding rate $=\frac{\text{D}}{\text{D}\;+\;\text{opponents}\;\text{O}}$
**F.oEFG** opponents EFG $=\frac{\text{opponents}\;\text{P2M}+1.5\;\text{oppponents}\;\text{P3M}}{\text{opponents}\;\text{P2A}\;+\;\text{opponents}\;\text{P3A}}$
**F.oTOR** opponents TOR $=\frac{\text{opponents}\;\text{TOV}}{\text{opponents}\;\text{NP}}$
**F.oFTR** opponents FTR $=\frac{\text{opponents}\;\text{FTM}}{\text{opponents}\;\text{P2A}\;+\;\text{opponents}\;\text{P3A}}$

The last 8 derived factors are based on the popular *Oliver’s four factors* for basketball [2, 26], 4 for the team and 4 for the opponent. For example, average F.EFG measures the team’s shooting effectiveness while average F.oEFG measures the team’s ability to limit the shooting effectiveness of other teams. Note that F.DRB is the same as opponents F.ORB (or F.oORB) but we use this more common notation instead. The main purpose Oliver’s four factors is to summarize the quality of team performance. They have the desirable properties of being practically orthogonal and practically independent of pace (that is, they are normalized for number of possessions, which makes them easier to compare across teams and competitions).

We also included for each game and each team the factor **Entropy.MIN**. This factor is the information-theoretic (Shannon) entropy of play time across players:

$$-\sum_{i=1}^{12}p_{i}\;\text{log}\;p_{i},$$

where

$$\begin{array}{c} p_{i}=\frac{\text{i}-\text{th}\;\text{players}\;\text{minutes}}{\text{total}\;\text{minutes}}. \end{array}$$

Entropy is a measure of uncertainty. In this case we use it to measure uncertainty in play time assignment, which is a proxy for the amount of player rotation.

### Statistical analysis

We analysed the data using R [25] and packages ggplot2 [27] and reshape2 [28].

#### Analysis of differences and trends

We performed an exploratory analysis of trends by plotting the variables over time, broken down by competition, regular season vs playoffs, and home vs away. To simplify interpretation, we added loess smoothing and plotted the standard errors.

We also fit a statistical model to the data to better quantify the means and trends and compare the NBA with the Euroleague, regular season with playoffs and playing at home with playing away from home.

Let *t*_1_ = 2000, …, *t*_17_ = 2016 be the seasons and *y*_*i*,*j*_ the observations of a variable, where *i* = 1‥*n* is the observation index and *j* = 1‥17 is the index of the season of that observation. Within the season, we model the observations with a normal distribution with season mean *μ*_*j*_ and season variability *σ*_*j*_. Between seasons, we allow for a linear trend:

$$\begin{array}{cc} y_{\cdot ,j} & ∼N(\mu_{j},\sigma_{j})\\ \mu_{j} & ∼N(\mu +s\left(t_{j}-2008\right),\sigma ), \end{array}$$

where *s* is the slope parameter, *μ* is the overall mean (centred on season 2008/09) and *σ* is the between-season variability of season means. We Stan’s default flat priors on *μ*, *σ*, *σ*., and *s*. We fit this model independently to each subgroup of interest and we performed post-hoc between-group comparisons of *μ* and *s* through their posterior distributions.

We performed Bayesian inference using Stan [29] and the RStan [30] package for R. To reduce MCMC estimation errors to practically negligible levels, we ran each model for 10000 sampling iterations (1000 warmup iterations). The results did not indicate any potential issues with convergence or mixing for any of the subgroups.

#### Post-hoc discrimination between winning and losing teams

We used a linear regression model to explain the variability in team’s win percentage with the average values of the 8 factors based on Oliver’s four factors. We measured model-fit with adjusted R squared. Note that we included other factors (one-by-one) but none of the other factors contributed information beyond what was already in the 8 factors. This holds for both leagues and all seasons.

We performed the analysis for (a) each competition and season separately, (b) each competition separately across all seasons, (c) both competitions combined across all seasons and (d) for the NBA and each season separately, but sub-sampling (with replacement) to the size of the Euroleague data for that season. The latter was relevant to exploring whether Oliver’s four factors explain less variability in the Euroleague or if the difference is only due to a smaller number of games and therefore greater variability in win percentages in the Euroleague. We did not aggregate team data for analyses that span over more than one season—each team-season pair was treated as a separate team.

## Results and discussion

In the following subsections we show and discuss the results of the statistical analyses. Note that the visual summaries for total counts and visual and numerical summaries for the home-away comparisons are omitted for brevity. Complete results are provided as supplementary material S1 Figs and S1 Tables.

### Changes over time

#### Pace

**Fig 1:**
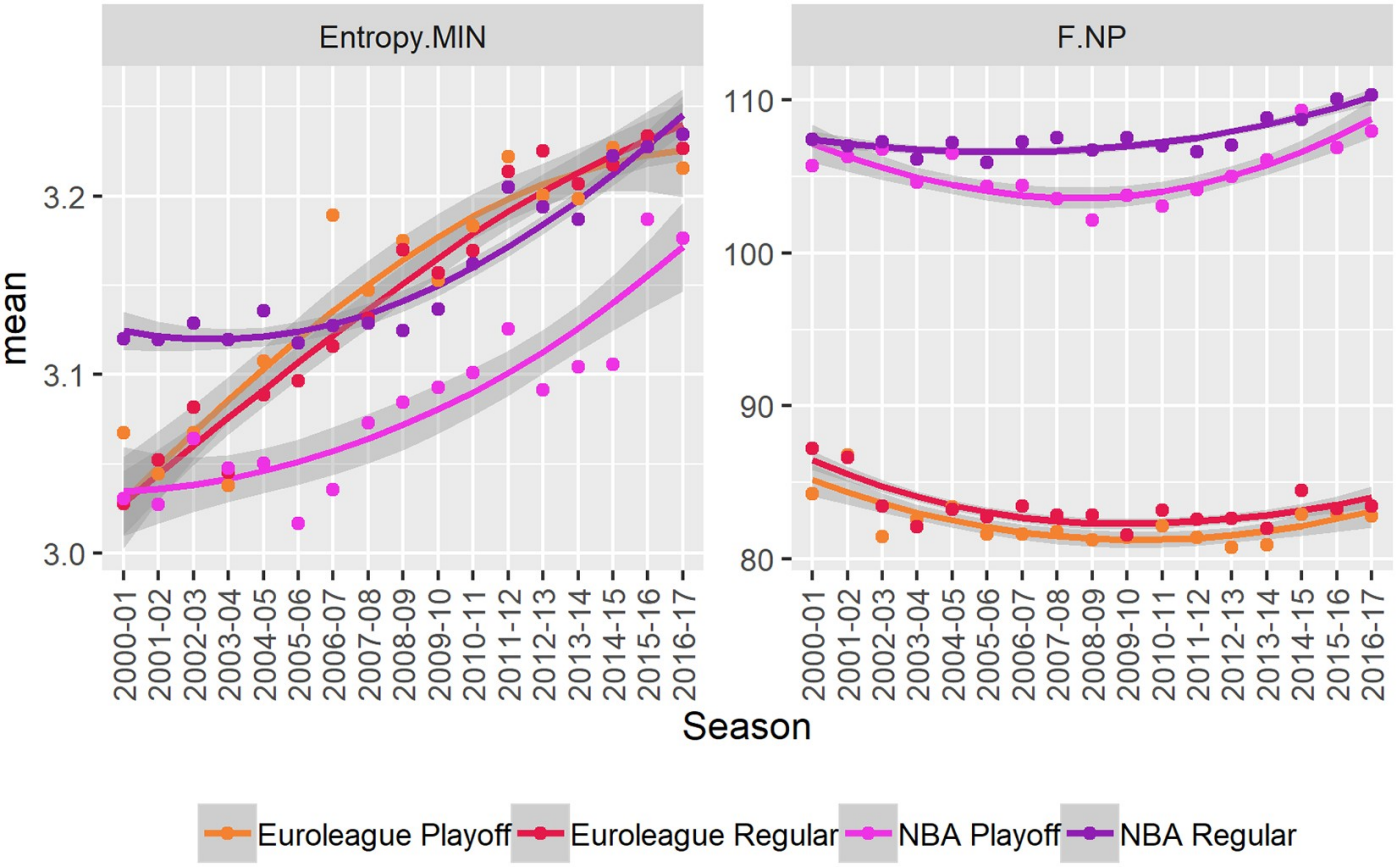
Changes over time. A visual summary of changes over time in player rotation (Entropy.MIN) and number of possessions per game (F.NP). The points indicate seasonal averages. The lines and shaded areas are loess smoothed individual (per game) observations with standard errors. Results are broken down by stage (regular season vs playoffs).

The number of possessions per game (F.NP) is significantly higher in the NBA (see Fig 1 and Table 1). A difference in the number of possessions is expected, due to a longer quarter (12 minutes in the NBA versus 10 minutes in the Euroleague). However, the ratio 107.4 ± 0.3 (NBA) over 83.0 ± 0.4 (Euroleague) is approximately 130% and exceeds the expected 120%. That is, the difference cannot be accounted for only by differences in game length, which implies a faster pace in the NBA. Therefore, the pace of the game (even after adjusting for playing time) is quicker in the NBA.

**Table 1:** A numerical comparison of the Euroleague and the NBA (regular season and playoffs combined). The first two columns are the estimated means (*μ*), followed by estimated difference in means (Δ_*μ*_), and posterior probability that the difference is positive ($P_{\Delta_{\mu }>0}$). The fifth and sixth column are the estimates slopes (*s*), followed by estimated difference in slope (Δ_*s*_), and posterior probability that the difference is positive ($P_{\Delta_{s}>0}$). Posterior probabilities under 0.01 or over 0.99 are marked with *. The second line for each variable contains the standard errors for the estimates. Note that the posterior distributions of *μ* and *s* are approximately normal so ± 2 standard errors is approximately the 95% Bayesian posterior confidence interval. Note that all estimates except for F.NP and Entropy.MIN are multiplied by 100 to simplify interpretation.

	*μ*_Euro_	*μ*_NBA_	Δ_*μ*_	$P_{\Delta_{\text{s}}>0}$	s_Euro_	s_NBA_	Δ_s_	$P_{\Delta_{\text{s}}>0}$
F.NP	83.00	107.41	-24.41	*0.000	-0.15	0.16	-0.31	*0.001
	0.36	0.26	0.44		0.07	0.05	0.09	
Entropy.MIN	3.15	3.15	-0.01	0.175	0.01	0.01	0.01	*1.000
	0.00	0.01	0.01		0.00	0.00	0.00	
F.P3pct	35.59	35.16	0.43	0.924	-0.00	0.03	-0.04	0.263
	0.26	0.15	0.30		0.05	0.03	0.06	
F.P2pct	51.84	48.12	3.72	*1.000	-0.06	0.22	-0.28	*0.000
	0.19	0.18	0.26		0.04	0.04	0.05	
F.FTpct	73.33	75.61	-2.28	*0.000	0.17	0.06	0.11	0.967
	0.23	0.18	0.29		0.05	0.04	0.06	
PaceAdj.Tot.FTA	24.72	22.61	2.11	*1.000	-0.50	-0.17	-0.32	*0.000
	0.19	0.26	0.32		0.04	0.05	0.06	
PaceAdj.Tot.P2A	46.90	58.67	-11.76	*0.000	-0.01	-0.46	0.45	*1.000
	0.35	0.26	0.44		0.07	0.05	0.09	
PaceAdj.Tot.P3A	24.55	17.17	7.38	*1.000	0.32	0.60	-0.28	*0.000
	0.26	0.28	0.38		0.05	0.06	0.08	
PaceAdj.Tot.STL	9.77	7.03	2.75	*1.000	-0.35	-0.01	-0.34	*0.000
	0.34	0.07	0.34		0.07	0.01	0.07	
PaceAdj.Tot.AST	16.79	20.05	-3.26	*0.000	0.47	0.02	0.45	*1.000
	0.23	0.09	0.25		0.05	0.02	0.05	
PaceAdj.Tot.BLK	3.12	4.58	-1.47	*0.000	0.04	-0.02	0.06	*1.000
	0.03	0.05	0.06		0.01	0.01	0.01	
PaceAdj.Tot.TOV	16.17	12.85	3.31	*1.000	-0.06	-0.05	-0.00	0.469
	0.18	0.08	0.19		0.04	0.02	0.04	
F.FTperF	92.79	113.81	-21.01	*0.000	-1.14	0.13	-1.27	*0.000
	0.56	0.66	0.86		0.11	0.13	0.17	
PaceAdj.Tot.FCM	26.38	19.75	6.63	*1.000	-0.19	-0.17	-0.02	0.323
	0.14	0.15	0.21		0.03	0.03	0.04	
F.DRB	70.00	73.52	-3.52	*0.000	0.03	0.30	-0.27	*0.000
	0.16	0.16	0.22		0.03	0.03	0.05	
F.ORB	30.01	26.48	3.53	*1.000	-0.03	-0.30	0.27	*1.000
	0.14	0.16	0.22		0.03	0.03	0.04	
PaceAdj.Tot.TRB	39.54	39.22	0.33	0.945	0.28	0.01	0.27	*1.000
	0.16	0.13	0.21		0.03	0.03	0.04	

The average number of possessions is relatively consistent throughout the observed period for both competitions. There is a significant negative trend in the Euroleague, attributable to the initial 2 seasons, which can be explained by the switch from 30s to 24s shot clock in 2000 which resulted in more fast-paced basketball until teams gradually adjusted to take full advantage of the 24s. There is also a significant positive trend in the NBA, which can be explained by a series of changes to off-ball fouls, clear-path fouls and similar changes which are aimed to quicken the flow of the game.

#### Player rotation

The amount of player rotation (Entropy.MIN) has been consistently increasing in recent years (see Fig 1). The positive trend is significant and significantly higher in the Euroleague. However, there is no significant difference in mean entropy in the observed period (see Table 1). The increase in player rotation can be explained by the increasing demands of top-tier competitive basketball. The intensity of the game requires more frequent rotation and distribution of playing time amongst players.

To aid in the interpretation of the entropy values of minutes played note that playing with only 5 players for the entire game would result in an entropy of approximately 2.3 bits. Giving each of the 12 players an equal number of minutes would result in an entropy of approximately 3.6 bits. Entropy of 3.2 bits is equivalent to rotating (equally) 9 players.

#### Shooting

**Fig 2:**
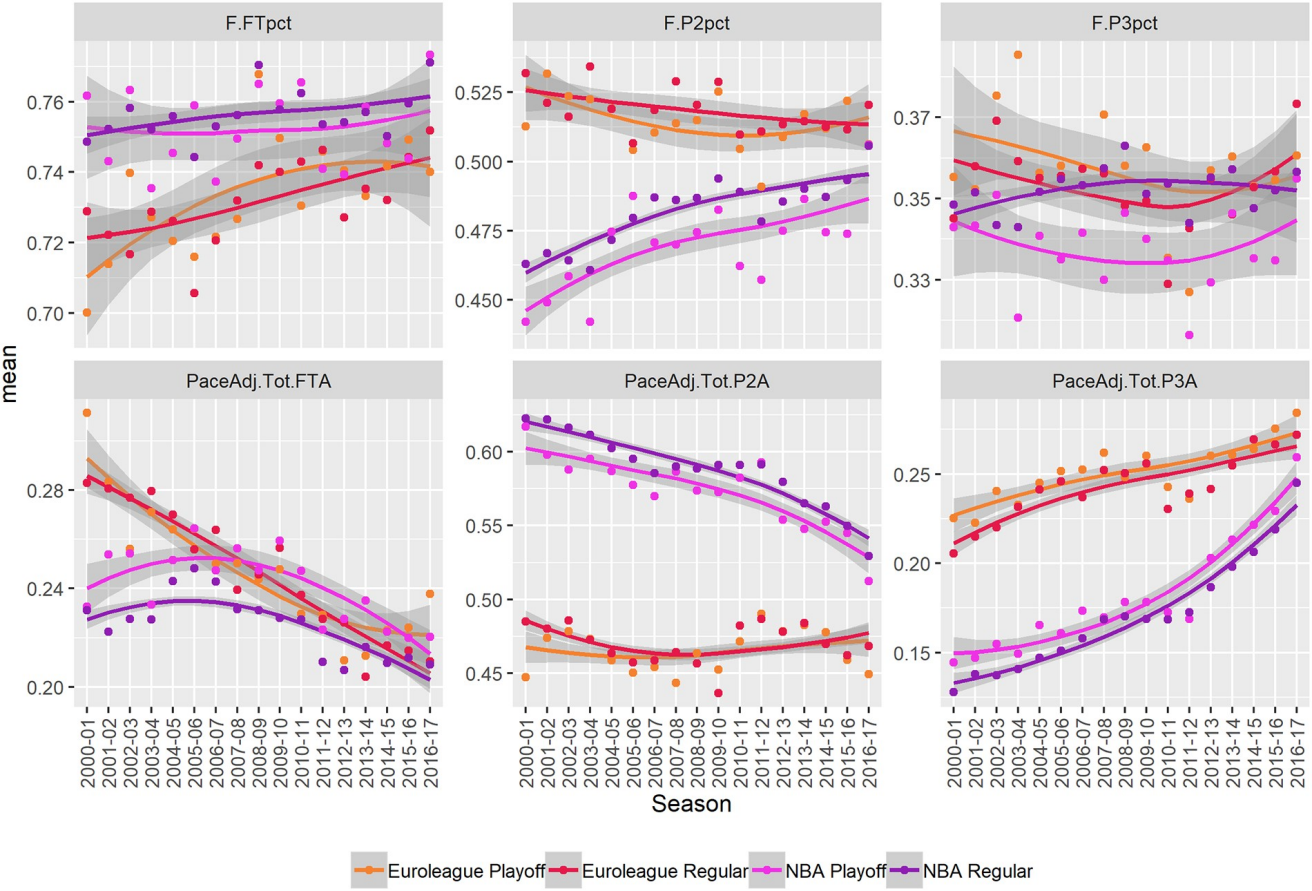
Changes over time. A visual summary of changes over time in free-throw (FT), two-point (P2), and three-point (P3) shooting percentages (pct) and shots attempted (PaceAdj.Tot). The points indicate seasonal averages. The lines and shaded areas are loess smoothed individual (per game) observations with standard errors. Results are broken down by stage (regular season vs playoffs).

The shooting related variables are visualized in Fig 2 and the numerical estimates of mean and trend are shown in Table 1. Free-throw% (F.FTpct) are higher in the NBA and the difference is estimated to be around 2.3%. Free-throw shooting is one of the few parameters where we can make a direct and absolute comparison of NBA and Euroleague players. The free-throw line distance is the same in both competitions and has not changed since 1895. Furthermore, free-throws are executed in standard and stable conditions, unobstructed and independently of other players. Looking further back to the 40s and 50s of the 20th century, we can see that players made 65 do 70% free throws [31]. In the same period, European players were less successful—the five best teams of the 1947 European national tournament finals in Prague made, on average, 50% free–throws. After that, free-throw percentages improved and the difference between NBA and European teams decreased. The current levels of free-throw shooting were reached in the NBA as far back as the 70s [31]. At around that time, the best 12 teams on the 1971 European championship in Germany made 66% of free-throws.

The number of free-throws attempted (PaceAdj.Tot.FTA) has been significantly decreasing in both competitions, but significantly more so in the Euroleague. While the number was higher in the Euroleague over the entire observed period, the difference more decreasing trend in the Euroleague has lead to very similar levels in both competitions in the most recent seasons. The decrease in free-throw attempts can be explained by the increasing free-throw percentages, in particular, a total disappearance of players that are really poor free-throw shooters. Subsequently, with 75% free-throw shooting, a personal foul that leads to free-throw attempts is, on average, worth 1.5 points per possession. Compared to the average value of a two-point shot (around 1 point per possession) and a three-point shot (around 1.1 points per possession), free-throws are by far the most effective. The logical tactical response is to reduce the number of opponent’s free-throws and only give personal fouls while they do not lead to free-throw attempts.

Note that when a team commits more than 4 fouls in a quarter (that is, when in bonus), the fifth and every subsequent team foul leads to 2 free-throws for the opposing team. Shooting fouls (that is, fouls committed on a player while he was attempting a shot) are an exception—a shooting foul always leads to 2 free-throws (or 1 free-throw if the fouled player made the shot).

On average, two-point % (F.P2pct) are higher in the Euroleague (+3.7%) and the number of two-point shots attempted (PaceAdj.P3A) is lower (-11.8 shots per 100 possessions). This can be attributed to shorter and less worked out offense in the NBA, however, there is a significant trend of decreasing number of two-point shot attempts and increasing two-point% in the NBA, while the Euroleague exhibits no significant trend in either.

The possessions that no longer end in two-point attempts in the NBA are replaced by three-point attempts. Without changes to the three-point arc, the number of three-point attempts (PaceAdj.Tot.P3A) has almost doubled in the NBA in the observed period. This indicates a substantial shift in how the game is played in the NBA. A similar trend of more three-point shots is observable in the Euroleague as well but the magnitude is half smaller. The increased number of three-point attempts is due to fewer free-throw attempts as the number of two-point attempts was consistent throughout the period.

The number of three-point shots attempted is higher in the Euroleague (+7.3 shots per 100 possessions). In terms of shooting percentages (F.P3pct) there are no significant differences in mean or trend. Although the three-point arc is 0.5m further away in the NBA and NBA players are more athletic defenders, this result is not surprising. NBA players attempt fewer three-point shots per possession, which implies better shot selection, NBA players are, on average, better shooters and the more distant arc requires players to defend a substantially larger perimeter in the NBA.

#### Assists, blocks, steals, turnovers

**Fig 3:**
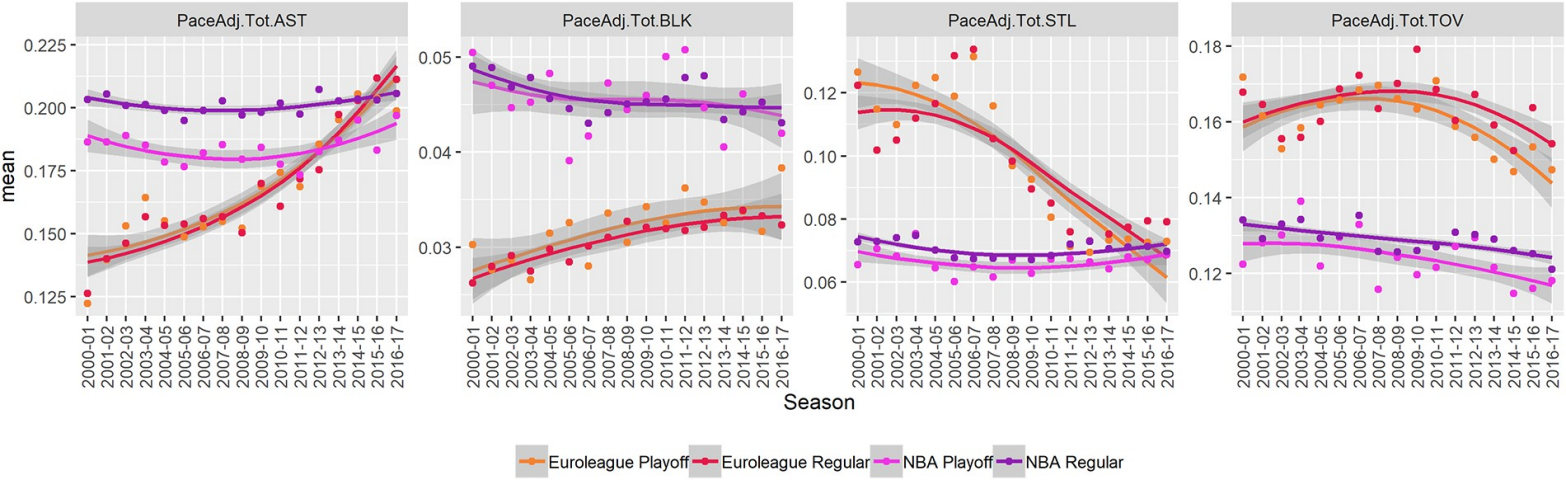
Changes over time. A visual summary of changes over time in the number of assists (PaceAdj.Tot.AST), blocks (PaceAdj.Tot.BLK), steals (PaceAdj.Tot.STL), and turnovers (PaceAdj.Tot.TOV). The points indicate seasonal averages. The lines and shaded areas are loess smoothed individual (per game) observations with standard errors. Results are broken down by stage (regular season vs playoffs).

Assists (PaceAdj.Tot.AST), blocks (BLK), steals (STL), and turnovers (TOV) are visualized in Fig 3 and the numerical estimates of mean and trend are shown in Table 1.

There are significant differences in all four variables in the observed period. Compared to the NBA, the Euroleague has fewer assists (-3.3) and blocks (-1.5), and more steals (+2.7) and turnovers (+3.3 per 100 possessions). However, there are significant trends in the Euroleague in assists, blocks, and steals that bring the levels closer to NBA levels (there are no significant trends in the NBA). There is a similar slight downward trend in turnovers in both competitions.

When interpreting assists we have to take into account that assists are given somewhat subjectively and the NBA standard for an assist is more generous than the Euroleague standard. The higher number of blocks in the NBA can be attributed to elite physical tools of some NBA players. The number of steals is in recent seasons the same in the NBA and the Euroleague, after a substantial decrease in steals in the Euroleague in the middle of the observed period, which can be attributed to changes in refereeing (reach-in fouls, etc.) [21].

#### Personal fouls

**Fig 4:**
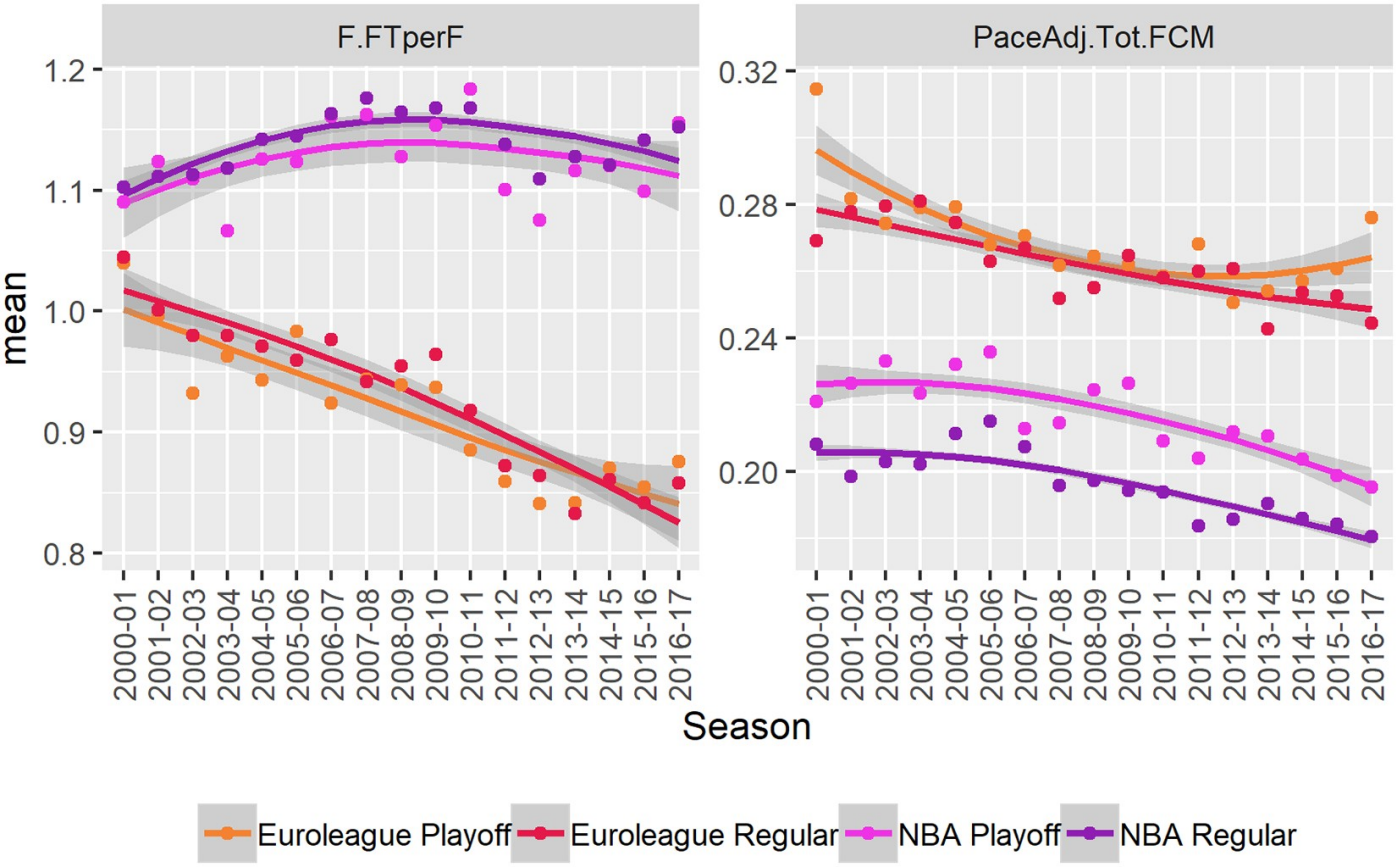
Changes over time. A visual summary of changes over time in the number of free-throws attempted per foul committed (F.FTperF) and the total number of fouls committed (PaceAdj.Tot.FCM). The points indicate seasonal averages. The lines and shaded areas are loess smoothed individual (per game) observations with standard errors. Results are broken down by stage (regular season vs playoffs).

Personal fouls committed (PaceAdj.Tot.FCM) and free-throws per foul (F.FTperF) are visualized in Fig 4 and the numerical estimates of mean and trend are shown in Table 1.

The number of fouls committed is higher in the Euroleague (+6.6 fouls per 100 possessions). There is no significant difference in trend—the number of fouls has been decreasing in both competitions at a rate of approximately 0.18 fouls per 100 possessions per season.

The results for the Euroleague give further support for our explanation of the decrease in the number of free throws. The number of fouls has decreased less than the number of free throws—the teams are becoming better at reducing the number of free throws per foul, making smarter fouls that do not lead to free throws (almost 0.2 free-throws less per foul over the entire period). There is no significant trend in the NBA and the number of free-throws per foul is much less efficient in the NBA compared to the Euroleague (0.2 free-throws more per foul).

#### Rebounding

**Fig 5:**
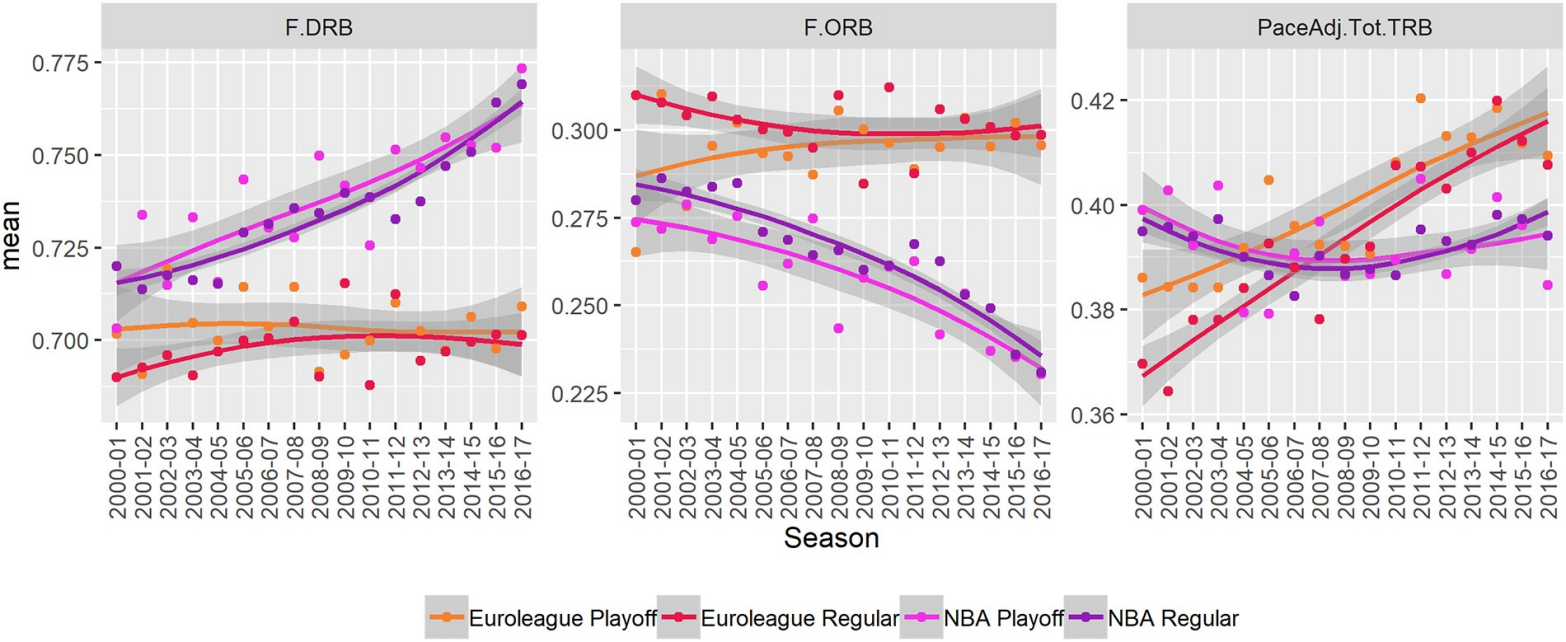
Changes over time. A visual summary of changes over time in the defensive (F.DRB) and the offensive (F.ORB) rebounding rates and the total number of rebounds (PaceAdj.Tot.TRB). The points indicate seasonal averages. The lines and shaded areas are loess smoothed individual (per game) observations with standard errors. Results are broken down by stage (regular season vs playoffs).

The rebounding variables are visualized in Fig 5 and the numerical estimates of mean and trend are shown in Table 1.

On average over the observed period the total number of rebounds (PaceAdj.Tot.TRB) is similar in both competitions. However, the number has been consistent in the NBA, while in the Euroleague there is a substantial positive trend. That is, the number of rebounds was much lower in the Euroleague at the start of the period but is now higher than in the NBA.

The defensive and offensive rebounding rates exhibit no trend in the Euroleague, while in the NBA, the defensive rebounding rate has been increasing throughout the period and is much higher than in the Euroleague (difference between 5% and 7% in recent seasons).

The difference in defensive rebounding rate is arguably not due to lack of effort, because it is the same in the playoffs, where the level of competitiveness increases. A gradual increase also rules out that it is only due to a change in refereeing criteria. A more plausible explanation is the elite rebounding of NBA defensive centers and that the risk associated with committing players to offensive rebounding is higher in the NBA. A major contributing factor is the athleticism and subsequent efficiency of some NBA players in transition, which often requires double-teaming to prevent them from scoring or drawing a foul on every transition.

#### Regular season vs. Playoffs

In this section we compare the regular season and playoffs across all variables. Visual summaries are available in Fig 1 through Fig 5 and the numerical summaries are provided in Tables 2 and 3, for the Euroleague and the NBA, respectively.

**Table 2:** A numerical comparison of the Euroleague regular season and Euroleague playoffs. The first two columns are the estimated means (*μ*), followed by estimated difference in means (Δ_*μ*_), and posterior probability that the difference is positive ($P_{\Delta_{\mu }>0}$). The fifth and sixth column are the estimates slopes (*s*), followed by estimated difference in slope (Δ_*s*_), and posterior probability that the difference is positive ($P_{\Delta_{s}>0}$). Posterior probabilities under 0.01 or over 0.99 are marked with *. The second line for each variable contains the standard errors for the estimates. Note that the posterior distributions of *μ* and *s* are approximately normal so ± 2 standard errors is approximately the 95% Bayesian posterior confidence interval. Note that all estimates except for F.NP and Entropy.MIN are multiplied by 100 to simplify interpretation.

	*μ*_Reg_	*μ*_Pla_	Δ_*μ*_	$P_{\Delta_{\text{s}}>0}$	s_Reg_	s_Pla_	Δ_s_	$P_{\Delta_{\text{s}}>0}$
F.NP	83.38	82.22	1.16	0.981	-0.13	-0.14	0.02	0.552
	0.38	0.39	0.55		0.08	0.08	0.11	
Entropy.MIN	3.14	3.15	-0.01	0.162	0.01	0.01	0.00	0.622
	0.00	0.01	0.01		0.00	0.00	0.00	
F.P3pct	35.39	35.96	-0.57	0.082	-0.01	-0.05	0.04	0.699
	0.28	0.30	0.41		0.06	0.06	0.09	
F.P2pct	51.87	51.75	0.11	0.640	-0.06	-0.07	0.01	0.532
	0.20	0.24	0.31		0.04	0.05	0.06	
F.FTpct	73.21	73.46	-0.26	0.261	0.16	0.17	-0.01	0.433
	0.25	0.35	0.43		0.05	0.07	0.09	
PaceAdj.Tot.FTA	24.64	24.95	-0.31	0.215	-0.51	-0.48	-0.03	0.377
	0.21	0.34	0.40		0.04	0.07	0.08	
PaceAdj.Tot.P2A	47.02	46.51	0.51	0.834	-0.03	0.03	-0.07	0.269
	0.37	0.38	0.53		0.08	0.08	0.11	
PaceAdj.Tot.P3A	24.29	25.11	-0.82	0.023	0.31	0.28	0.03	0.646
	0.28	0.29	0.40		0.06	0.06	0.08	
PaceAdj.Tot.STL	9.78	9.87	-0.09	0.418	-0.32	-0.45	0.13	0.914
	0.34	0.28	0.44		0.07	0.06	0.09	
PaceAdj.Tot.AST	16.71	16.91	-0.20	0.276	0.46	0.46	-0.00	0.499
	0.25	0.24	0.35		0.05	0.05	0.07	
PaceAdj.Tot.BLK	3.07	3.28	-0.21	*0.000	0.04	0.05	-0.01	0.190
	0.03	0.02	0.04		0.01	0.01	0.01	
PaceAdj.Tot.TOV	16.36	15.87	0.49	0.972	-0.03	-0.09	0.06	0.884
	0.20	0.16	0.26		0.04	0.03	0.05	
F.FTperF	93.02	92.08	0.94	0.861	-1.17	-1.05	-0.12	0.258
	0.57	0.67	0.88		0.12	0.14	0.18	
PaceAdj.Tot.FCM	26.22	26.85	-0.63	0.021	-0.19	-0.19	-0.01	0.449
	0.17	0.25	0.31		0.04	0.05	0.07	
F.DRB	69.85	70.44	-0.59	0.019	0.05	-0.05	0.10	0.963
	0.19	0.21	0.28		0.04	0.04	0.06	
F.ORB	30.15	29.58	0.58	0.975	-0.06	0.05	-0.10	0.045
	0.19	0.23	0.30		0.04	0.05	0.06	
PaceAdj.Tot.TRB	39.30	40.17	-0.88	*0.001	0.29	0.23	0.07	0.916
	0.17	0.17	0.24		0.03	0.04	0.05	

**Table 3:** A numerical comparison of the NBA regular season and NBA playoffs. The first two columns are the estimated means (*μ*), followed by estimated difference in means (Δ_*μ*_), and posterior probability that the difference is positive ($P_{\Delta_{\mu }>0}$). The fifth and sixth column are the estimates slopes (*s*), followed by estimated difference in slope (Δ_*s*_), and posterior probability that the difference is positive ($P_{\Delta_{s}>0}$). Posterior probabilities under 0.01 or over 0.99 are marked with *. The second line for each variable contains the standard errors for the estimates. Note that the posterior distributions of *μ* and *s* are approximately normal so ± 2 standard errors is approximately the 95% Bayesian posterior confidence interval. Note that all estimates except for F.NP and Entropy.MIN are multiplied by 100 to simplify interpretation.

	*μ*_Reg_	*μ*_Pla_	Δ_*μ*_	$P_{\Delta_{\text{s}}>0}$	s_Reg_	s_Pla_	Δ_s_	$P_{\Delta_{\text{s}}>0}$
F.NP	107.57	105.15	2.41	*1.000	0.17	0.10	0.07	0.708
	0.25	0.55	0.60		0.05	0.11	0.12	
Entropy.MIN	3.16	3.08	0.08	*1.000	0.01	0.01	-0.00	0.110
	0.01	0.01	0.01		0.00	0.00	0.00	
F.P3pct	35.20	34.50	0.70	0.985	0.03	0.05	-0.01	0.418
	0.14	0.29	0.32		0.03	0.06	0.06	
F.P2pct	48.16	47.53	0.64	0.952	0.22	0.25	-0.03	0.354
	0.18	0.34	0.39		0.04	0.07	0.08	
F.FTpct	75.63	75.32	0.31	0.792	0.06	0.08	-0.02	0.413
	0.17	0.34	0.38		0.04	0.07	0.08	
PaceAdj.Tot.FTA	22.49	24.31	-1.82	*0.000	-0.17	-0.20	0.03	0.641
	0.26	0.28	0.38		0.05	0.06	0.08	
PaceAdj.Tot.P2A	58.78	56.96	1.82	*1.000	-0.46	-0.46	-0.01	0.473
	0.26	0.39	0.47		0.05	0.08	0.09	
PaceAdj.Tot.P3A	17.08	18.55	-1.47	*0.002	0.60	0.62	-0.02	0.430
	0.26	0.42	0.49		0.05	0.08	0.10	
PaceAdj.Tot.STL	7.04	6.77	0.27	0.987	-0.01	0.00	-0.01	0.255
	0.07	0.09	0.11		0.01	0.02	0.02	
PaceAdj.Tot.AST	20.14	18.73	1.41	*1.000	0.02	0.04	-0.02	0.299
	0.09	0.19	0.21		0.02	0.04	0.04	
PaceAdj.Tot.BLK	4.57	4.74	-0.17	0.072	-0.02	-0.02	-0.00	0.494
	0.05	0.10	0.11		0.01	0.02	0.02	
PaceAdj.Tot.TOV	12.89	12.32	0.57	*1.000	-0.05	-0.06	0.01	0.659
	0.08	0.12	0.15		0.02	0.03	0.03	
F.FTperF	113.89	112.54	1.35	0.902	0.14	0.07	0.07	0.623
	0.64	0.81	1.04		0.13	0.17	0.21	
PaceAdj.Tot.FCM	19.63	21.44	-1.80	*0.000	-0.17	-0.18	0.01	0.562
	0.16	0.19	0.25		0.03	0.04	0.05	
F.DRB	73.49	74.00	-0.52	0.031	0.30	0.28	0.02	0.633
	0.17	0.22	0.28		0.03	0.04	0.06	
F.ORB	26.52	25.99	0.52	0.967	-0.30	-0.28	-0.02	0.357
	0.17	0.21	0.28		0.03	0.04	0.06	
PaceAdj.Tot.TRB	39.19	39.61	-0.42	0.044	0.01	-0.04	0.05	0.846
	0.13	0.21	0.25		0.03	0.04	0.05	

The numerical summaries for the Euroleague reveal that there are no statistically significant differences between the regular season and the playoffs, with the exception of the total number of blocks (+0.2 blocks per 100 possessions in the playoffs) and rebounds (+0.9 rebounds per 100 possessions per 100 possessions in the playoffs). And even these differences are practically very small.

There are no statistically discernible differences in trends between the regular season and the playoffs in either competition. That is, all the trends mentioned above are the same in both stages of the competition.

In the NBA there are more differences in means. The pace is slower in the playoffs (-2.4 possessions per game). There is less player rotation (-0.08 bits). There is fewer two-point attempts (-1.8 per 100 possessions) and more three-point (+1.5 per 100 possessions) and free-throw attempts (+1.8 per 100 possessions). There are also fewer assists (-1.4 per 100 possessions) and turnovers (-0.6 per 100 possessions) and more fouls committed (+1.8 per 100 possessions). With the exception of turnovers, all of these changes are in the direction of the Euroleague—that is, the NBA playoffs are more similar to the Euroleague than the NBA regular season.

#### Home team advantage

Visual and numerical summaries for the home-away comparison are provided as supplementary material. In this subsection we only briefly discuss. As expected, all statistically significant differences in all variables and both competitions are in favour of the home team. There are also no statistically significant differences in trends between the home and away teams.

The pattern of variables where the difference between home and away teams is not significant is almost identical for both competitions: number of possessions—this is expected, as the number of possessions of the home and away team is highly correlated (end of a possession implies the possession of the other team), player rotation—again, expected, because teams’ rotation depends more on the tactics than on playing home or away, free-throw percentage—the only set situation in a basketball game, and the number of two- and three-point attempts—again, home-away does not affect tactics. These results are consistent with the explanation that playing home or away does not significantly affect tactics, but it does significantly affect how effective teams are.

### Post-hoc discrimination between winning and losing teams

**Fig 6:**
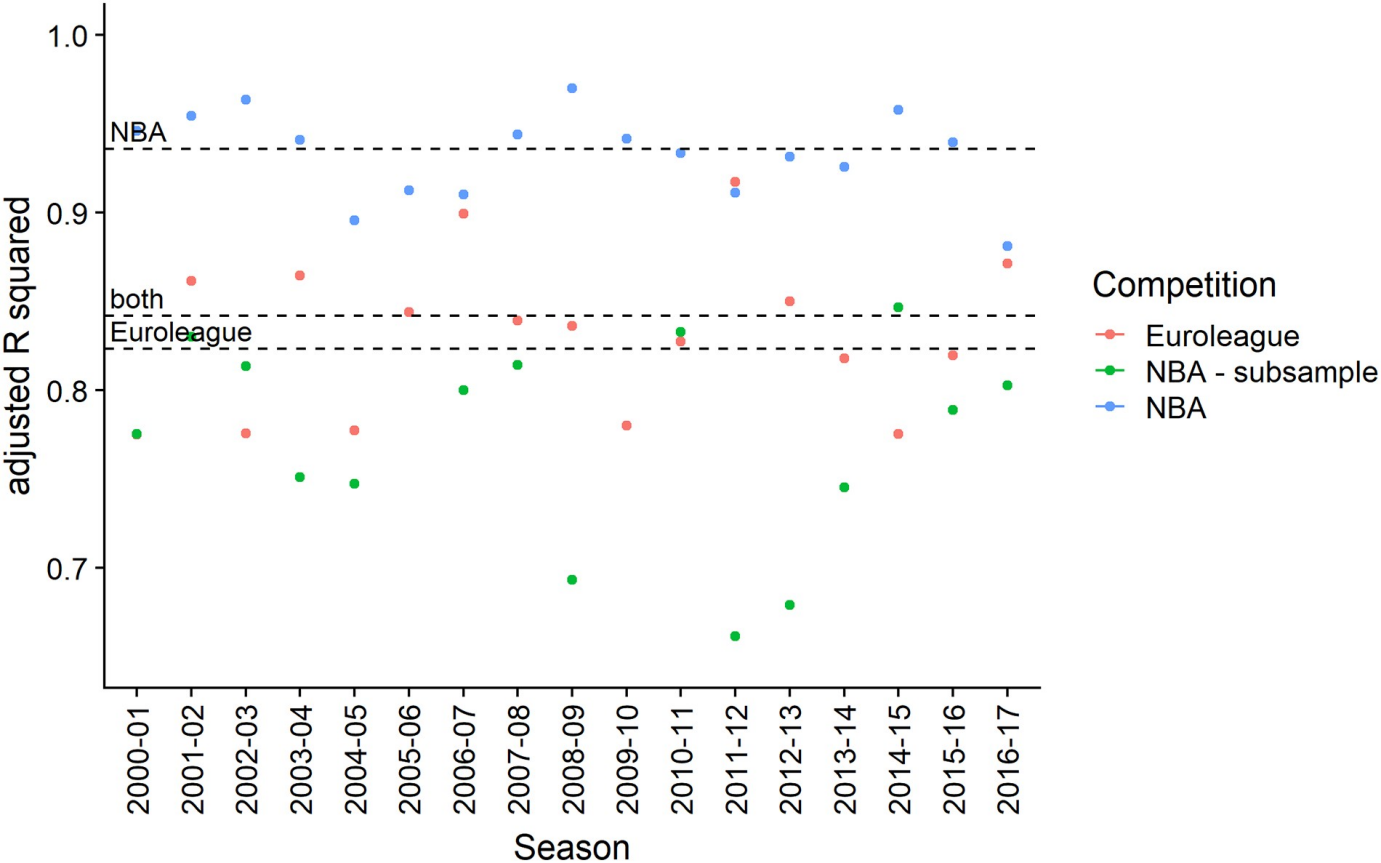
Linear model *R*^2^. A visual summary of the estimated adjusted R^2^ values for the linear prediction model of team success. The dashed lines represent the estimates across all seasons.

Results in Fig 6 provide insight into the relationship between Oliver’s four factors and win percentages. First, for the NBA, almost all of the variability in win percentages can be explained with four factor-based variables. And this holds not only for individual seasons, but also if a single model is fit for all seasons. This implies that the relationship remains consistent over time.

Second, in the Euroleague less variability in win percentages can be explained with the same variables. However, if we adjust the NBA results for the smaller number of games available in the Euroleague, we get similar results. That is, the discrepancy can be explained by the fact that Euroleague win percentages have more residual variability, because they are averages over a smaller sample size.

And third, even if the model is fit across all seasons simultaneously for NBA and Euroleague data, without identifying the competition, the adjusted R^2^ is close to the average of the two individual results. This implies that the relationship between the variables and win percentages is very similar for the two competitions. While it is not so surprising that for both competitions winning is a consequence of shooting effectively, not turning over many balls, forcing a lot of fouls and rebounding well (and forcing the opponent into doing these things poorly), it is somewhat more surprising that the weights of these factors are consistent not only across seasons but also across competitions.

The results are also a testament to the utility of Oliver’s four factors in providing a post-hoc explanation of team success. However, this does not imply that basketball outcomes are easy to predict or that four factor-based variables carry a lot of information for ex-ante predictions. On the contrary, there is evidence from many different competitions and time periods that basketball outcomes at the highest level of competition are difficult to predict. Even bookmaker odds (arguably the best publicly available source of probabilistic forecasts) can predict the outcome correctly in about 70% of games, while a home-team advantage is in most competitions between 60% and 70% (see [32] and references therein). Oliver’s four factors, while they do have some predictive power, are not able to outperform betting markets or even some more simple ranking-based models. So, while it is in the long run fairly easy to discriminate between good and bad teams, it is very difficult to predict the outcome of an particular game. In other words, the difference in quality between the best and worst teams in a competition is very small relative to the uncertainty that is inherent to basketball.

## Conclusion

The results of our analyses support the hypothesis that the highest level of European basketball—the Euroleague—is becoming quantitatively and qualitatively more similar to the NBA. There are no discernible differences in player rotation and, after adjusting for pace and accounting for trend, no substantial differences in assists, steals, or total rebounds and differences in two-point/three-point shooting patterns are decreasing. Blocks and structure of rebounds (that is, defensive rebounding rate) are the exception—both are substantially higher in the NBA. We hypothesize that blocks are the results of better physical tools of NBA defenders and that a higher rebounding rate is a tactical choice of not committing as many players to offensive rebounding in the NBA. Both hypotheses require further research. There are more personal fouls per possession in the Euroleague—a likely explanation is stricter refereeing rules and criteria or more aggressive play. The latter is consistent with the result that in the NBA playoffs the number of fouls increases. However, European players are better or more tactically prepared to reduce the number of free-throws per foul.

A major difference is in game pace. We would highlight this as the most evident difference between the two competitions. European basketball is more tactical (and becoming even more), based on longer positional and tactical offense, while the NBA has shorter possessions, less emphasis defense and tactical play and, as a consequence, more turnovers, transition play and, some would argue, more attractive basketball for the average viewer. Analysis of playoff data, however, reveals that NBA basketball becomes more similar to European basketball in the playoffs, which feature the best teams and a higher level of competitiveness. We argue that this is indirect evidence that NBA teams do not play at their highest level during the regular season, either to conserve strength or because winning is not the only imperative. However, once winning becomes more important, teams put more emphasis on tactical play and defence. This is something that European teams do more consistently, as there are no substantial differences between regular season and playoff games. Arguably, this could also be due to the fact that teams play fewer games in the Euroleague and individual games are more important.

The gradual decrease in differences between the NBA and the Euroleague is predominately due to rule changes. The remaining differences in games statistics will persist as long as there are differences in rules. The most substantial differences in rules that remain are 10 minute quarters, different 3-pt shooting arc (and slightly narrower/shorter court). However, there are also non-explicit differences in refereeing criteria. With statistics that are secondary to winning (points scored being the primary), such as assists, blocks, rebounds, etc., we also speculate that the differences have become smaller because there has been increasing emphasis in the Euroleague on players’ individual achievements in these categories, while in the past, the focus was predominately on points scored.

However, decreasing structural and quantitative differences as observed through game statistics, do not imply that the absolute difference in quality has also decreased. The absolute quality is more difficult to measure directly. Indirect evidence from international competitions, where team USA typically dominates, implies that the difference between top NBA players and top European players is still substantial. Another indirect indicator of this is purely financial. NBA median salary is at the level of Euroleague star players and NBA stars earn 10 times more, not including sponsorships. Arguably, most Euroleague players that are good enough to play in the NBA end up playing in the NBA.

## Supporting information

S1 Data The data used in the analysis. The data set is a R programming language data.frame stored as a serialized R object (see saveRDS()). The column names are consistent with the names used in this paper. Further information can be provided on request.
(ZIP)

S1 Figs Visual summaries. (ZIP)

S1 Tables Results tables. (ZIP)
