# Supplementary figures and images for: Trends in NBA and Euroleague basketball: Analysis and comparison of statistical data from 2000 to 2017
Source: PLoS One. 2019 Oct 7;14(10):e0223524. doi: 10.1371/journal.pone.0223524 (PMC6779240; doi:10.1371/journal.pone.0223524)

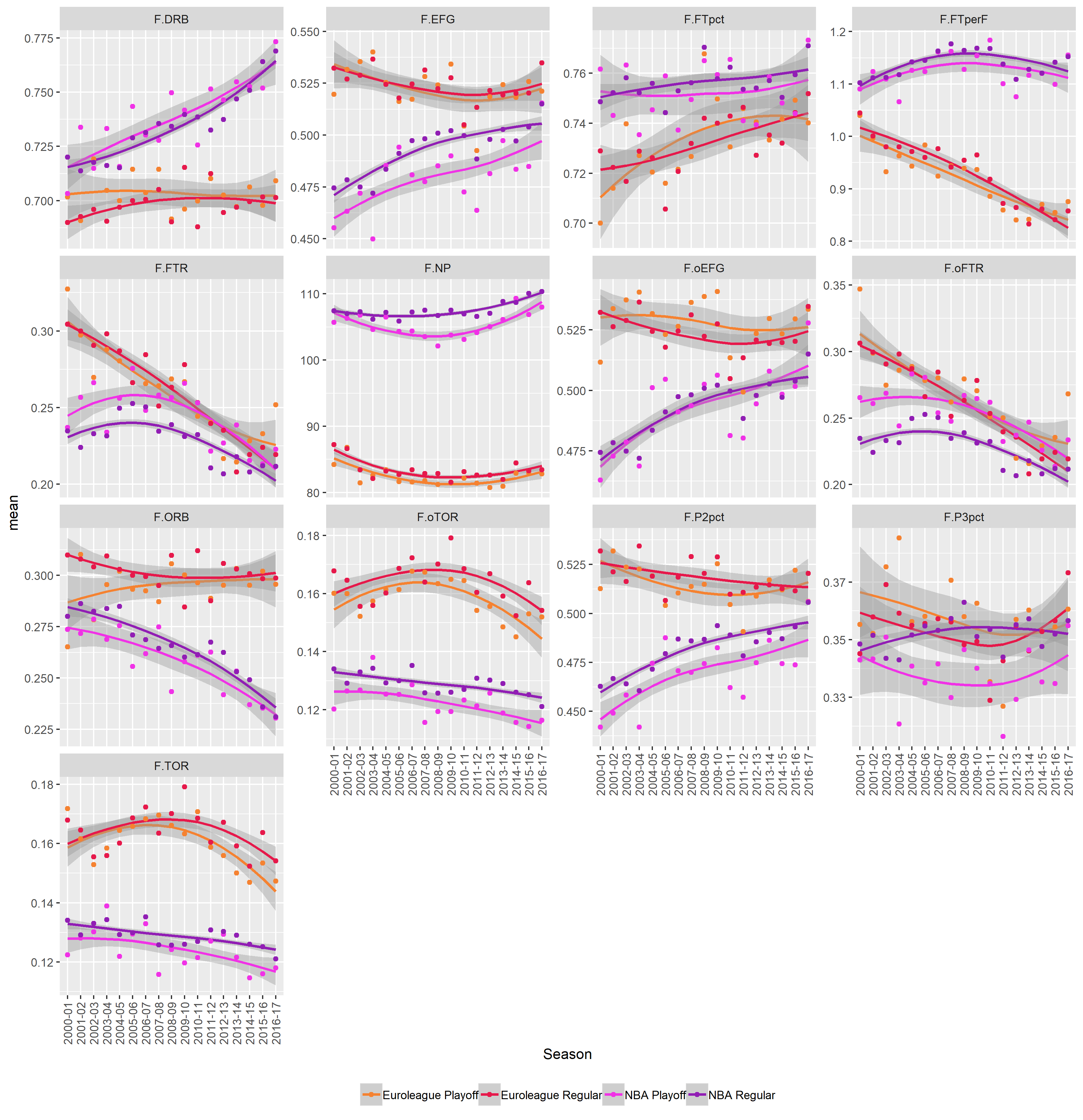

Supplement: S1 Figs — (ZIP) [file pone.0223524.s002.zip › all_f.tiff]

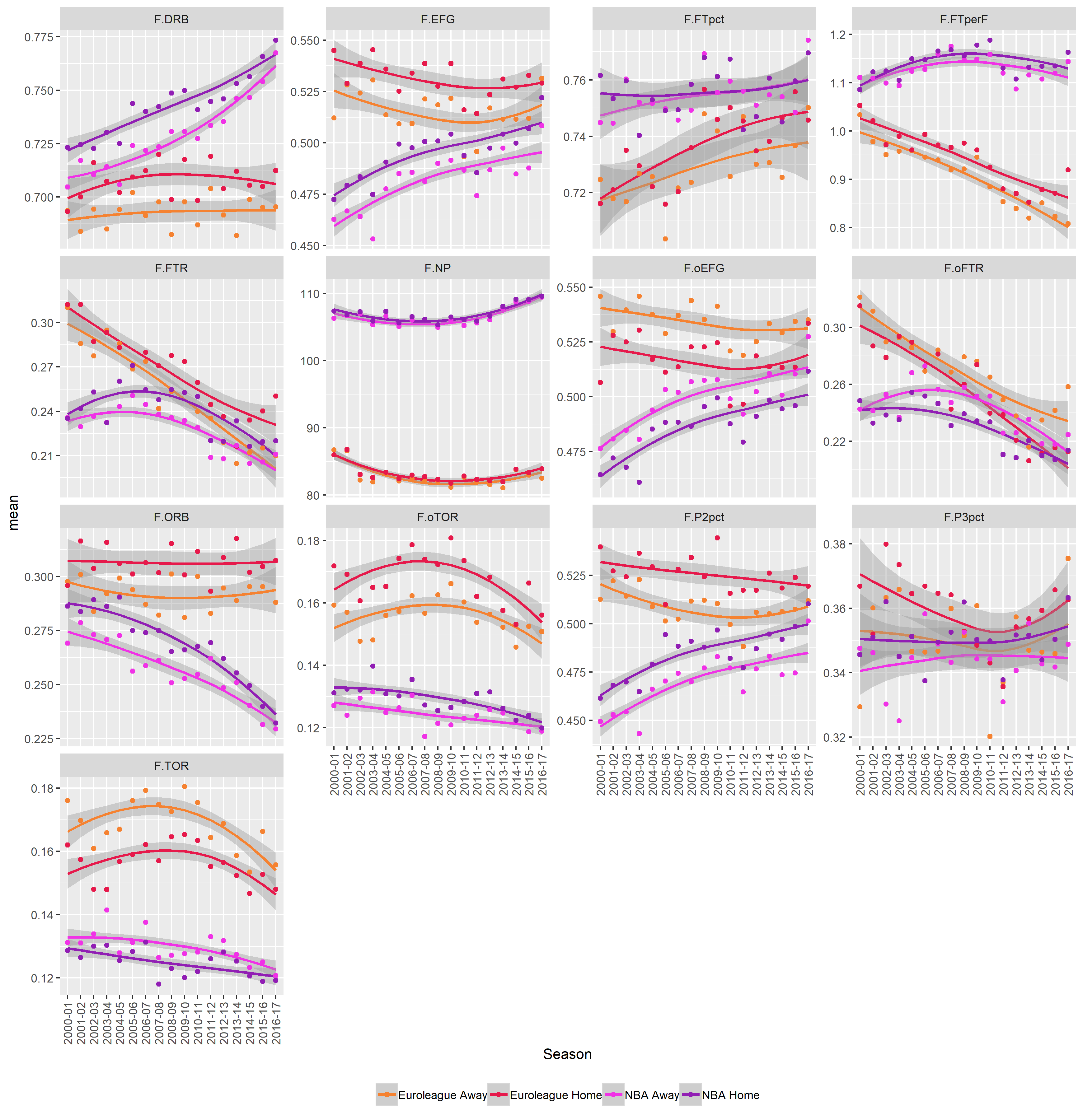

Supplement: S1 Figs — (ZIP) [file pone.0223524.s002.zip › all_f_home_team.tiff]

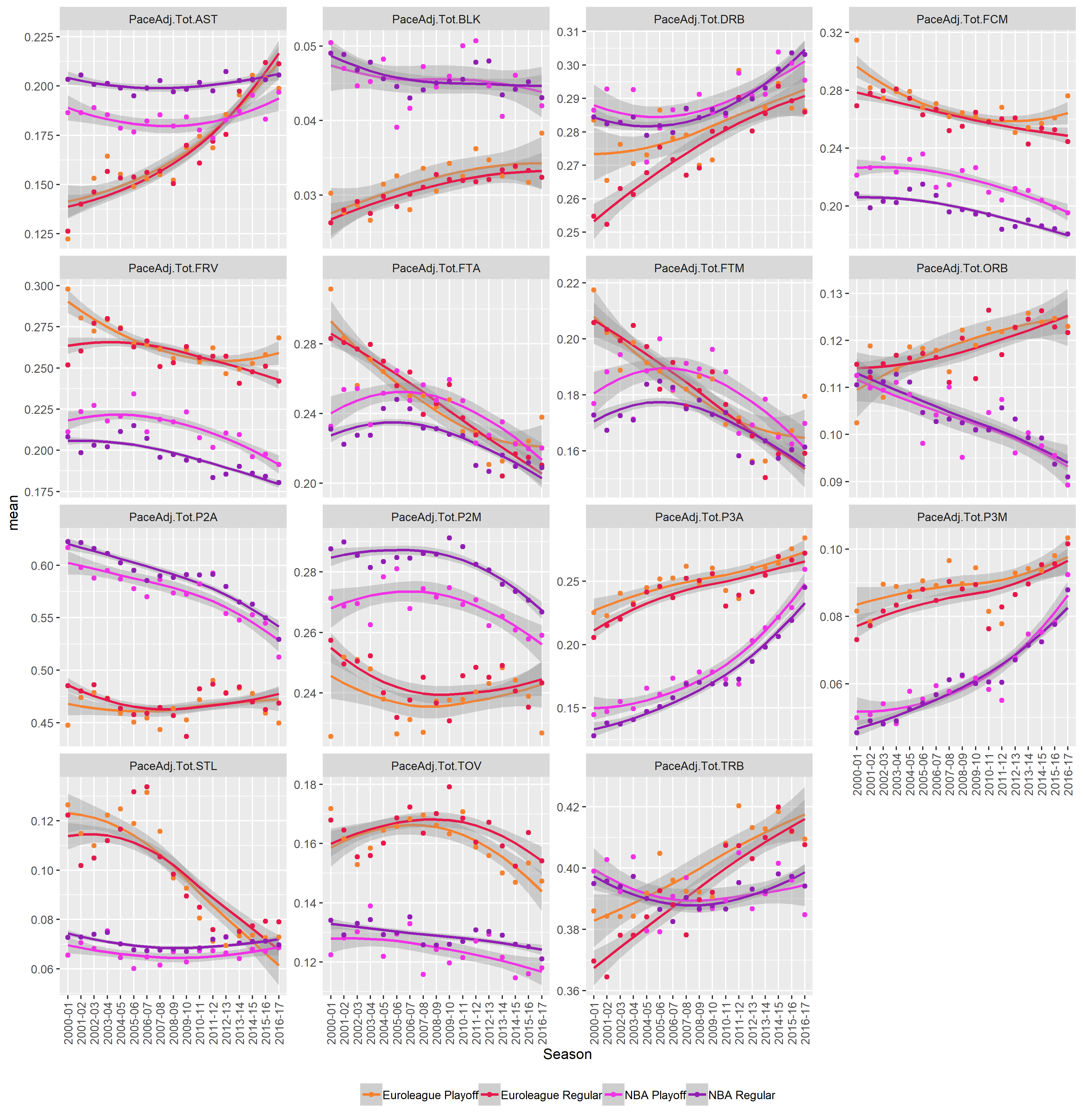

Supplement: S1 Figs — (ZIP) [file pone.0223524.s002.zip › all_paceadj.tiff]

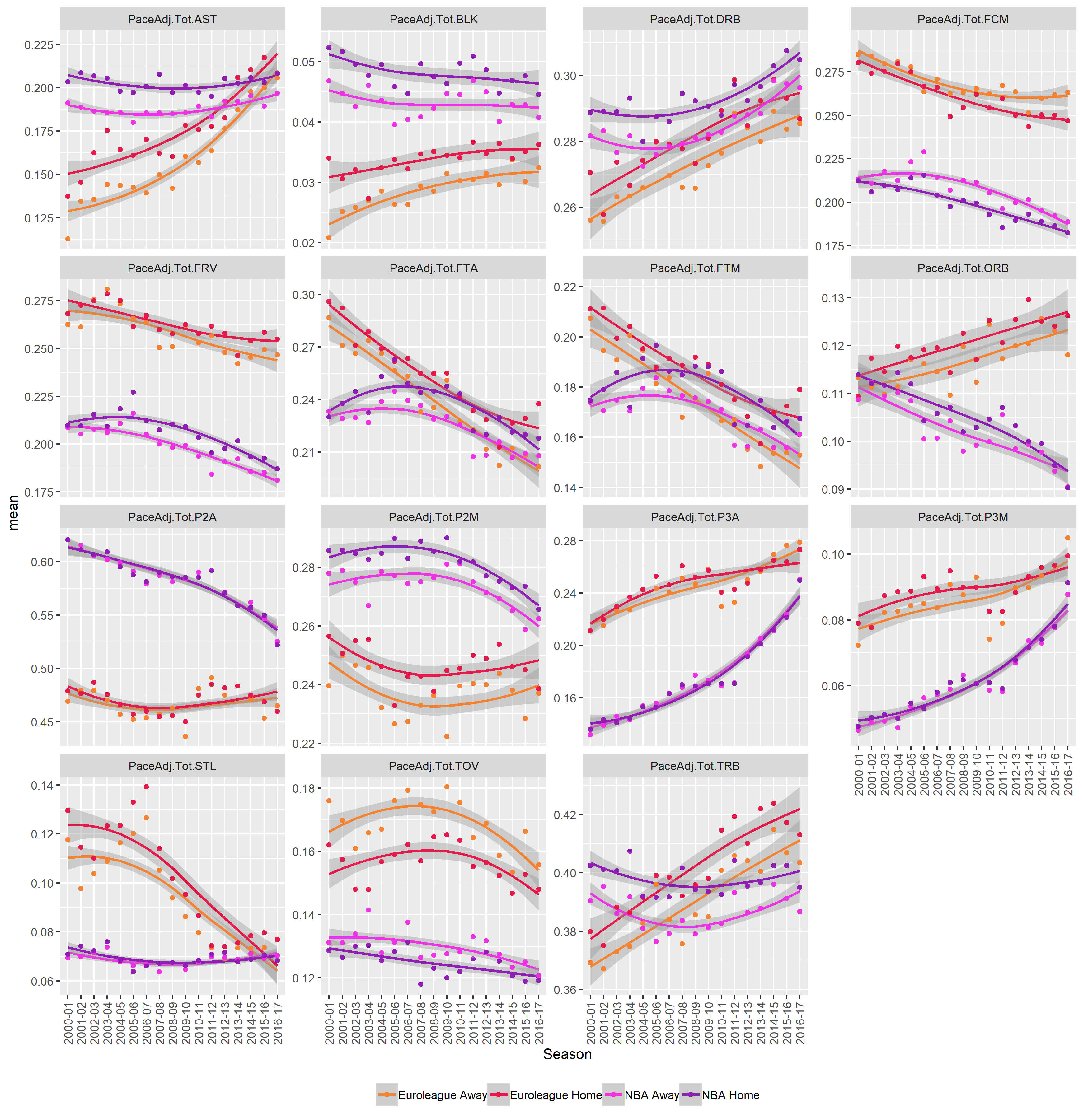

Supplement: S1 Figs — (ZIP) [file pone.0223524.s002.zip › all_paceadj_home_team.tiff]

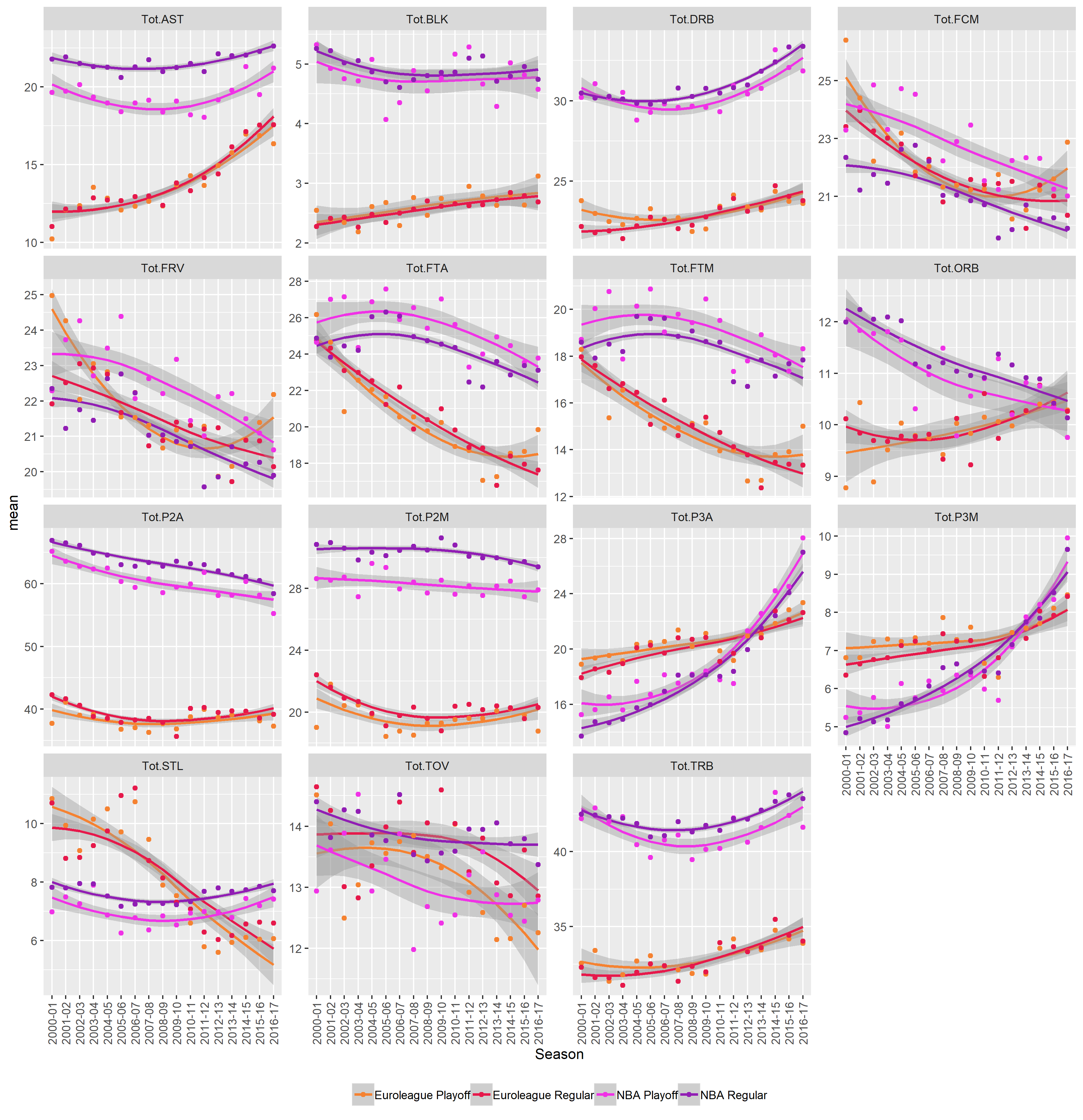

Supplement: S1 Figs — (ZIP) [file pone.0223524.s002.zip › all_tot.tiff]

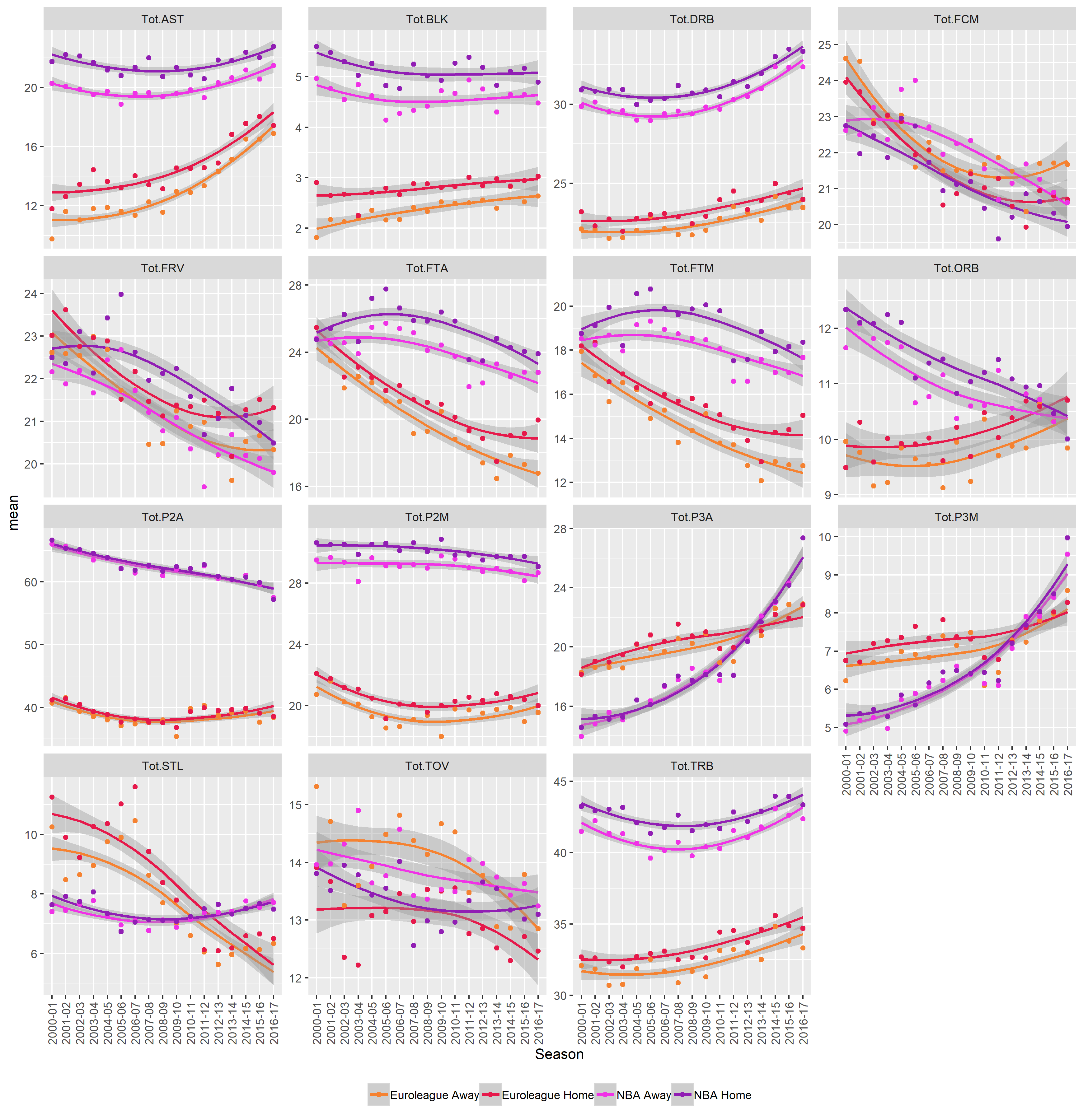

Supplement: S1 Figs — (ZIP) [file pone.0223524.s002.zip › all_tot_home_team.tiff]

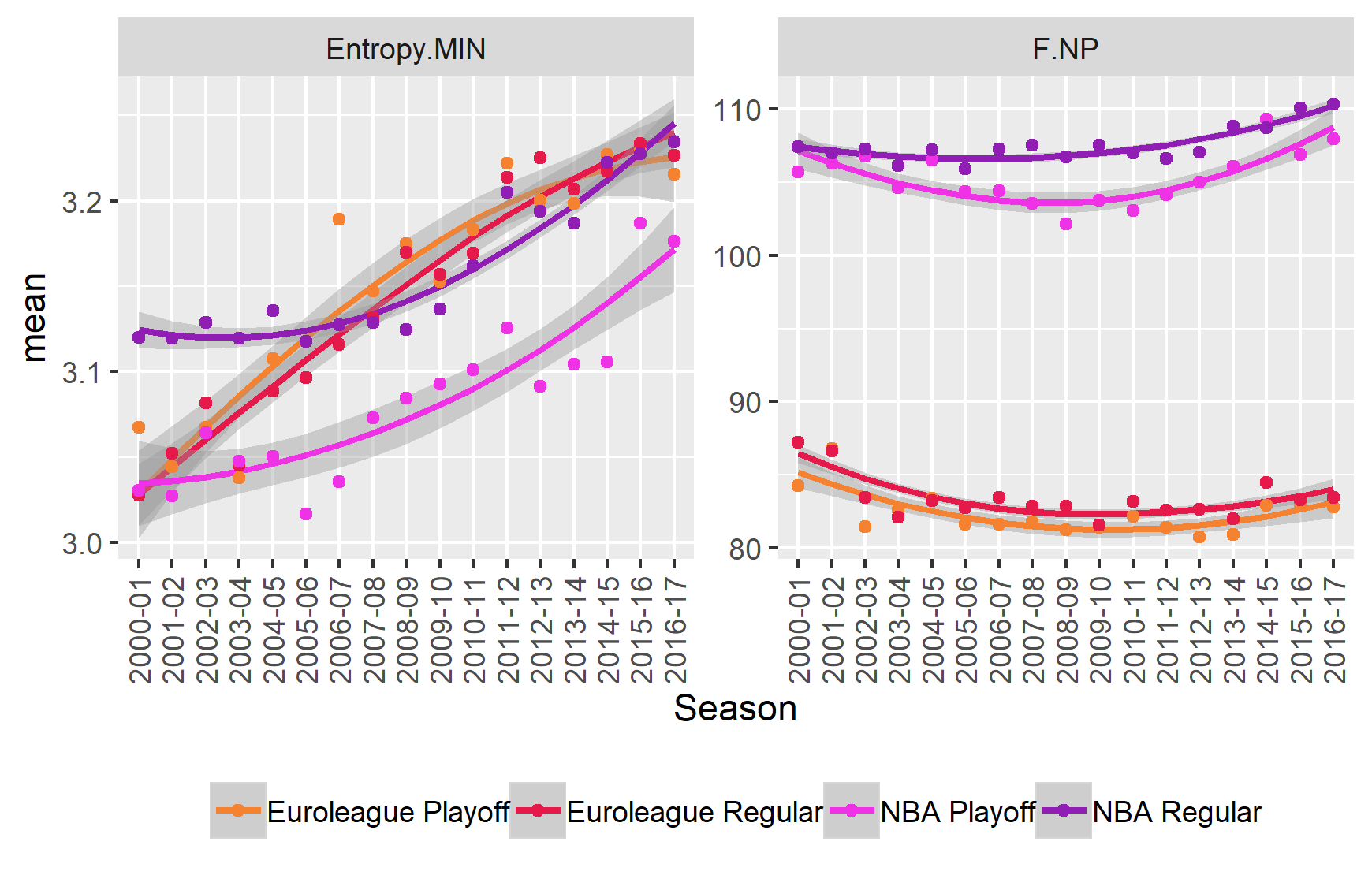

Supplement: S1 Figs — (ZIP) [file pone.0223524.s002.zip › Fig1.tiff]

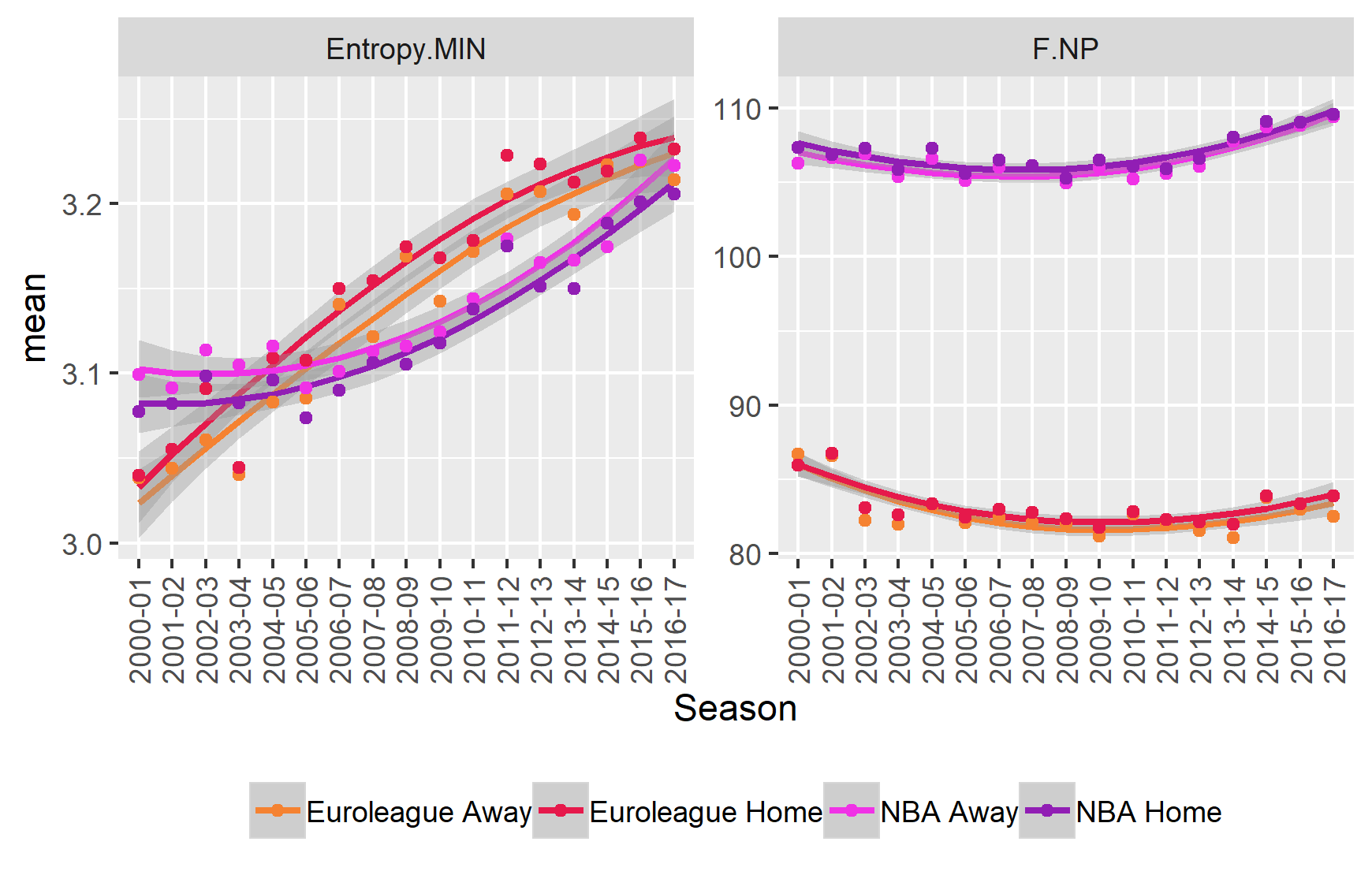

Supplement: S1 Figs — (ZIP) [file pone.0223524.s002.zip › Fig1_home_team.tiff]

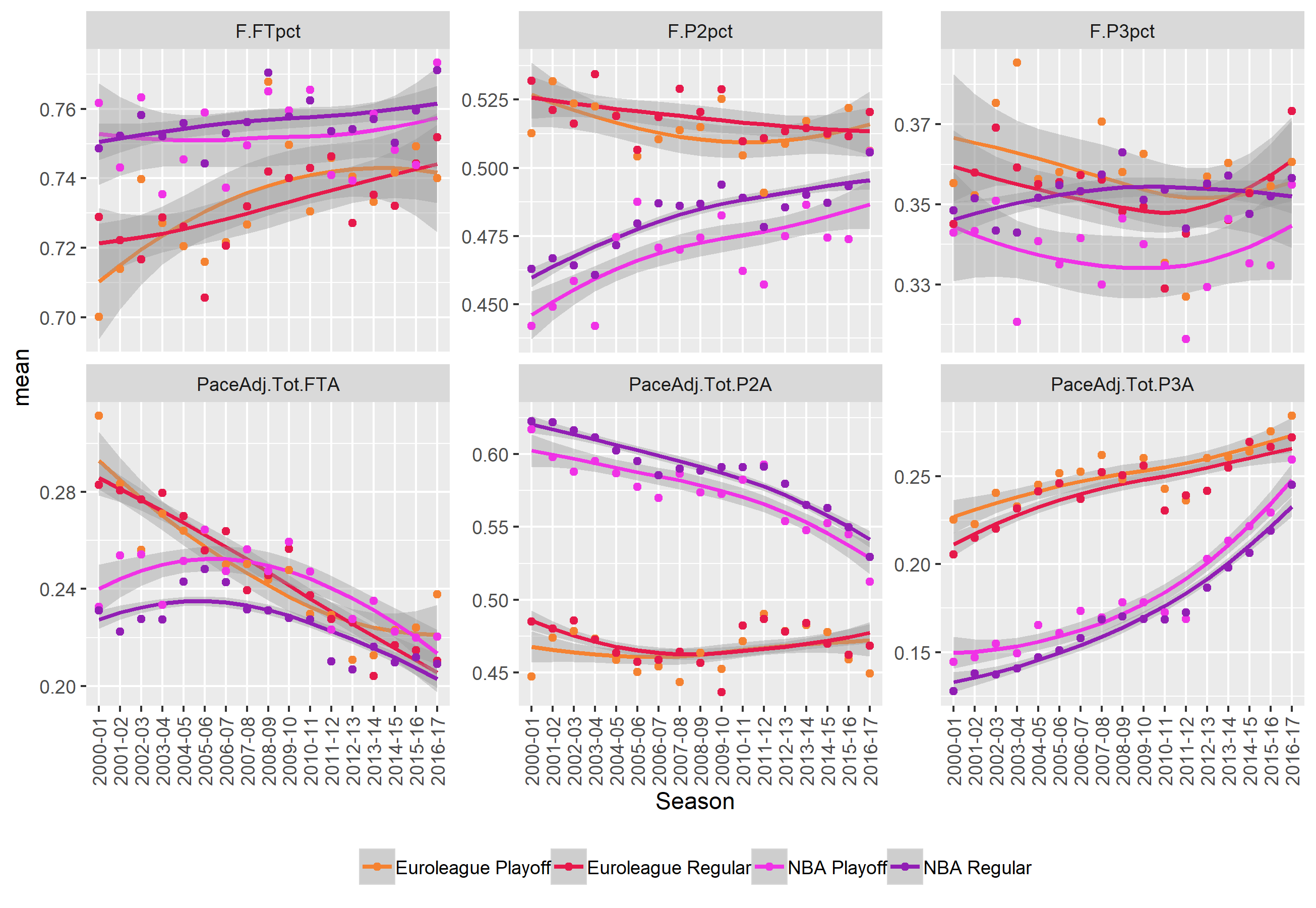

Supplement: S1 Figs — (ZIP) [file pone.0223524.s002.zip › Fig2.tiff]

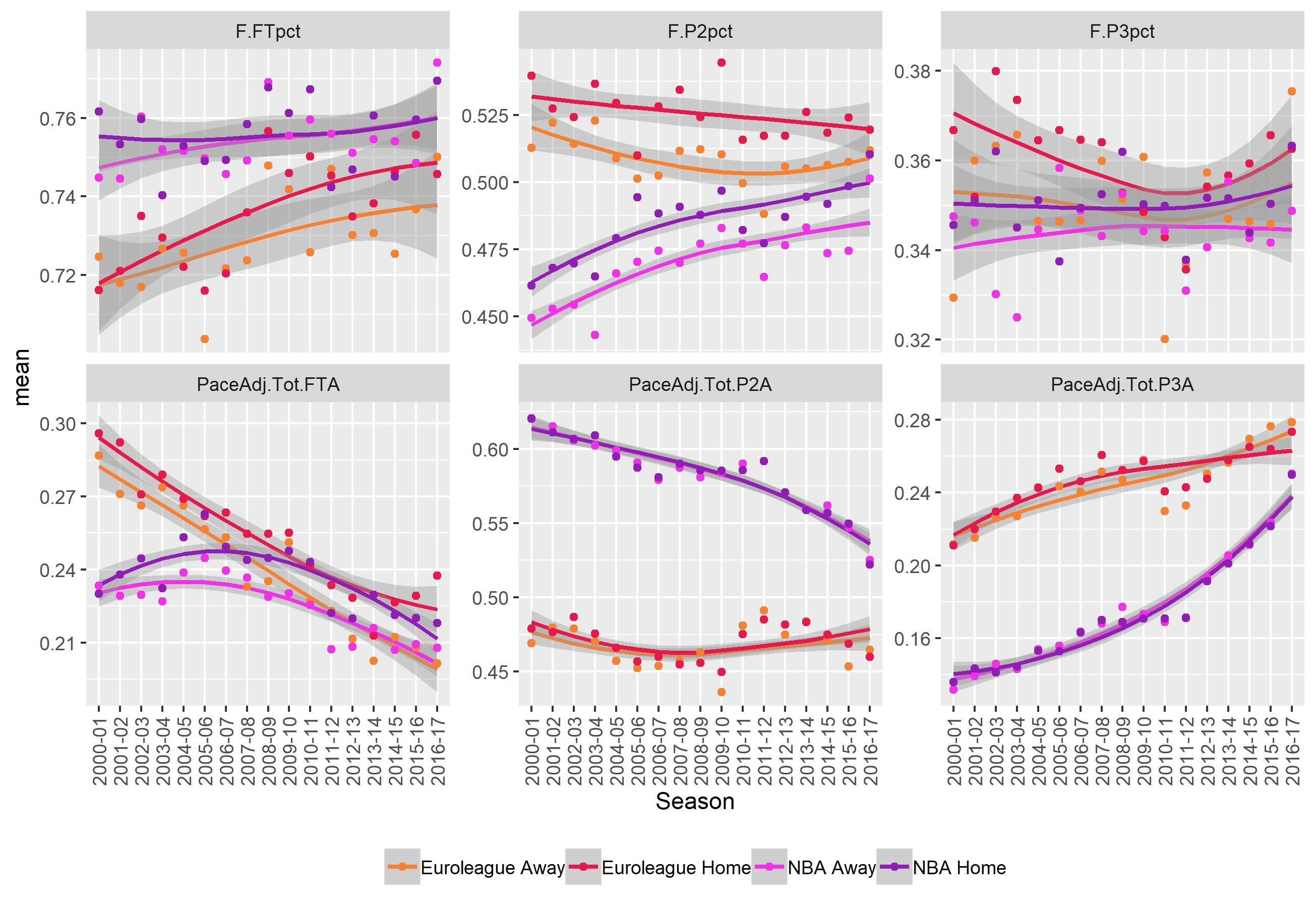

Supplement: S1 Figs — (ZIP) [file pone.0223524.s002.zip › Fig2_home_team.tiff]

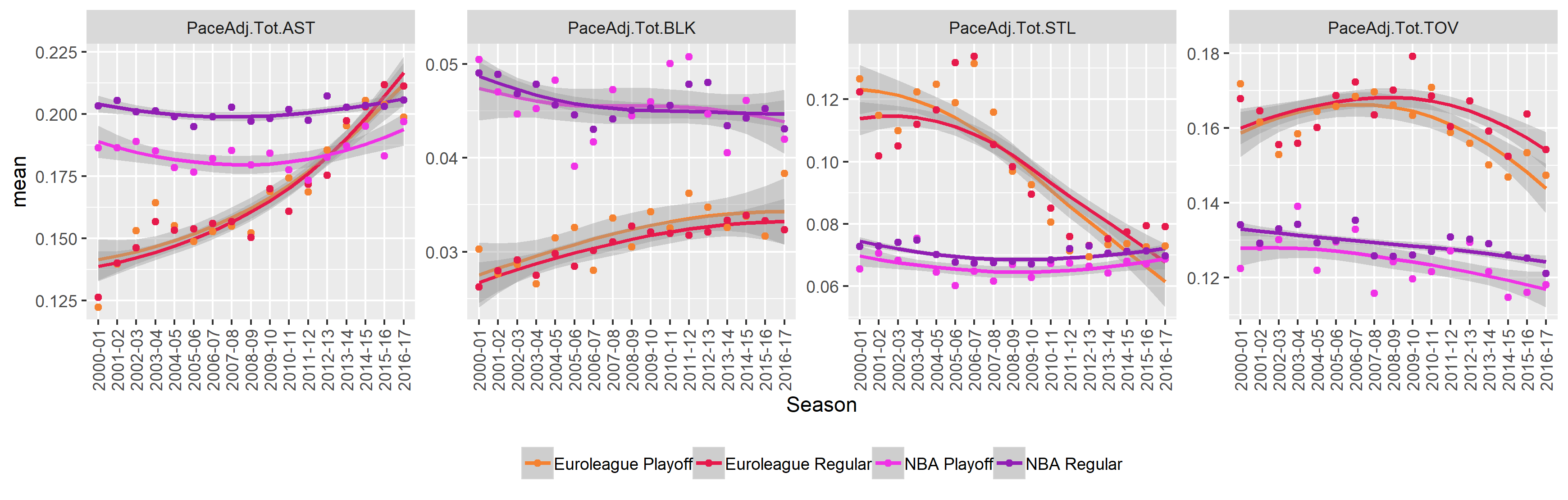

Supplement: S1 Figs — (ZIP) [file pone.0223524.s002.zip › Fig3.tiff]

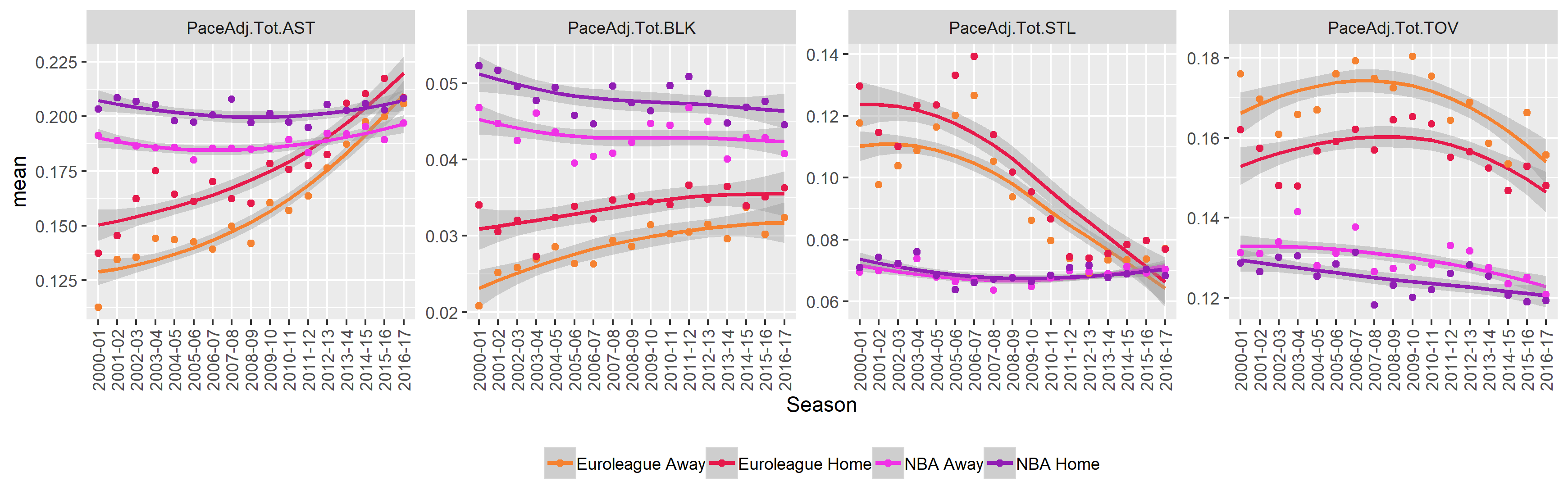

Supplement: S1 Figs — (ZIP) [file pone.0223524.s002.zip › Fig3_home_team.tiff]

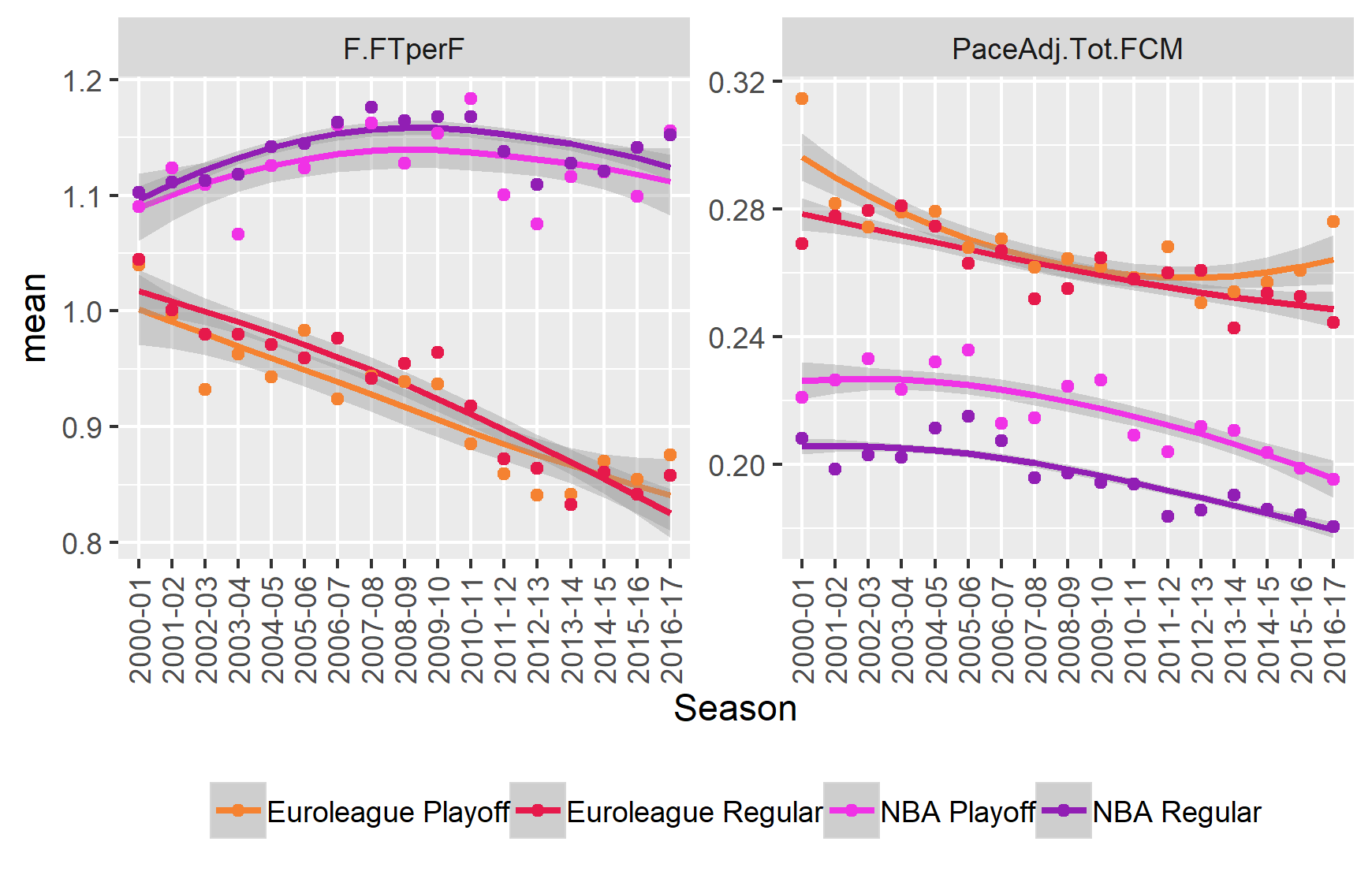

Supplement: S1 Figs — (ZIP) [file pone.0223524.s002.zip › Fig4.tiff]

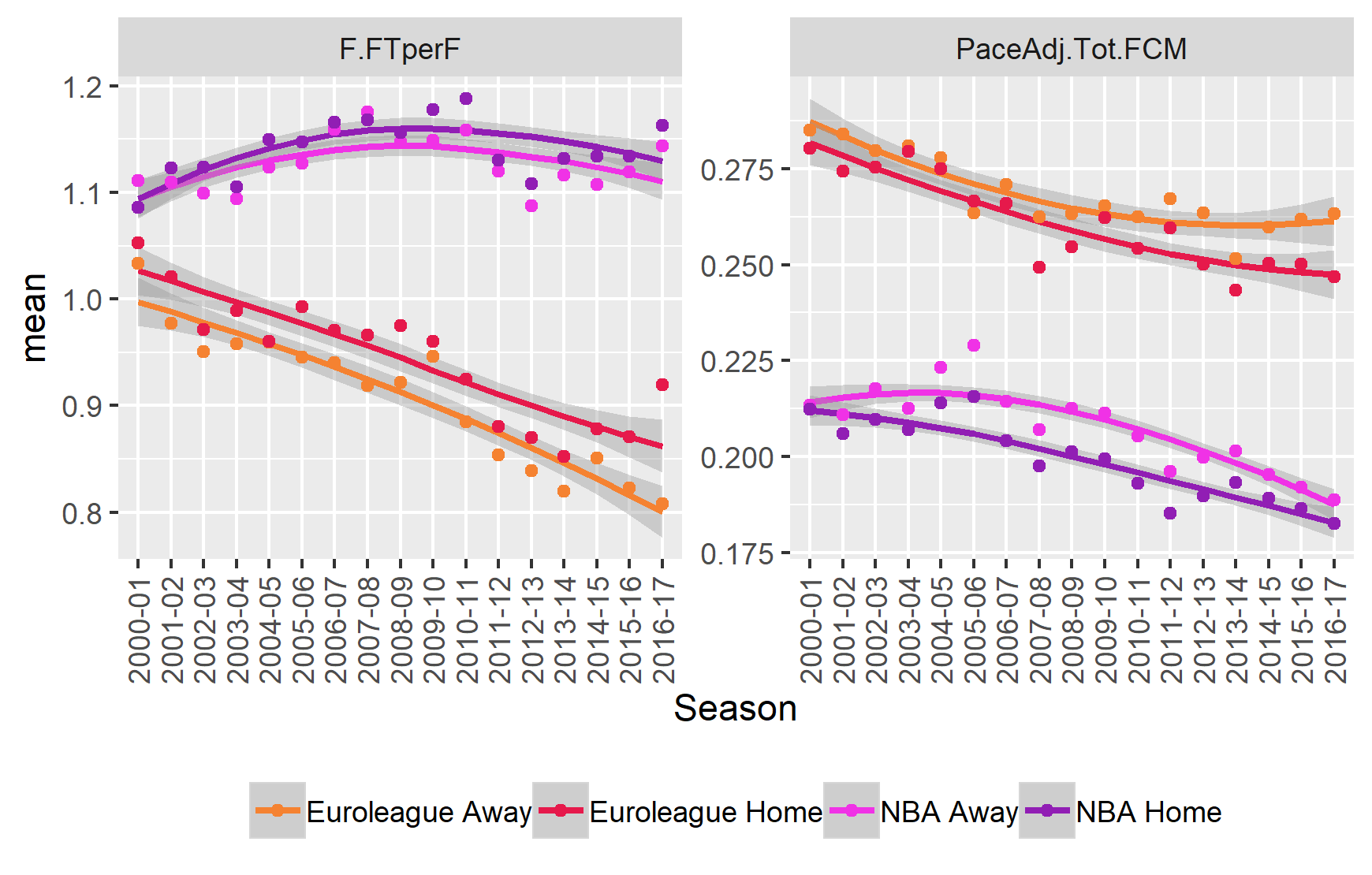

Supplement: S1 Figs — (ZIP) [file pone.0223524.s002.zip › Fig4_home_team.tiff]

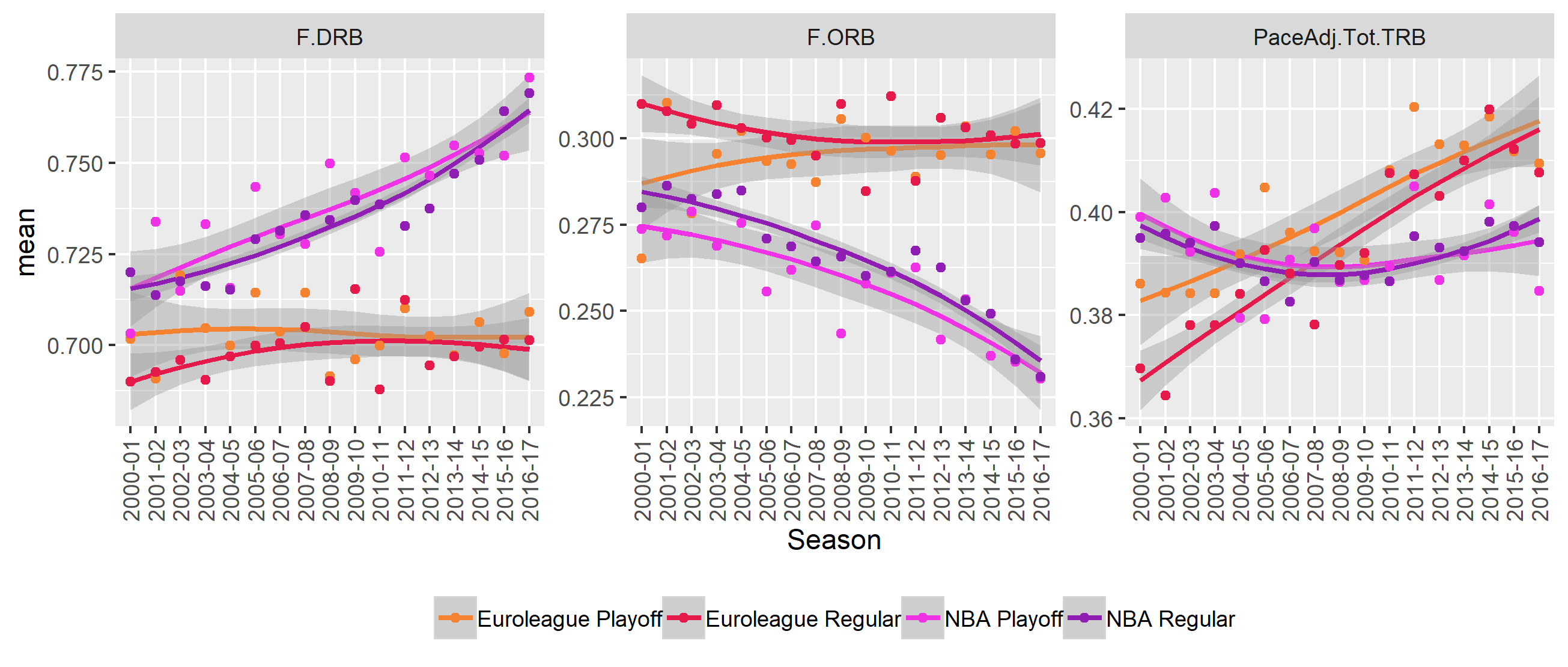

Supplement: S1 Figs — (ZIP) [file pone.0223524.s002.zip › Fig5.tiff]

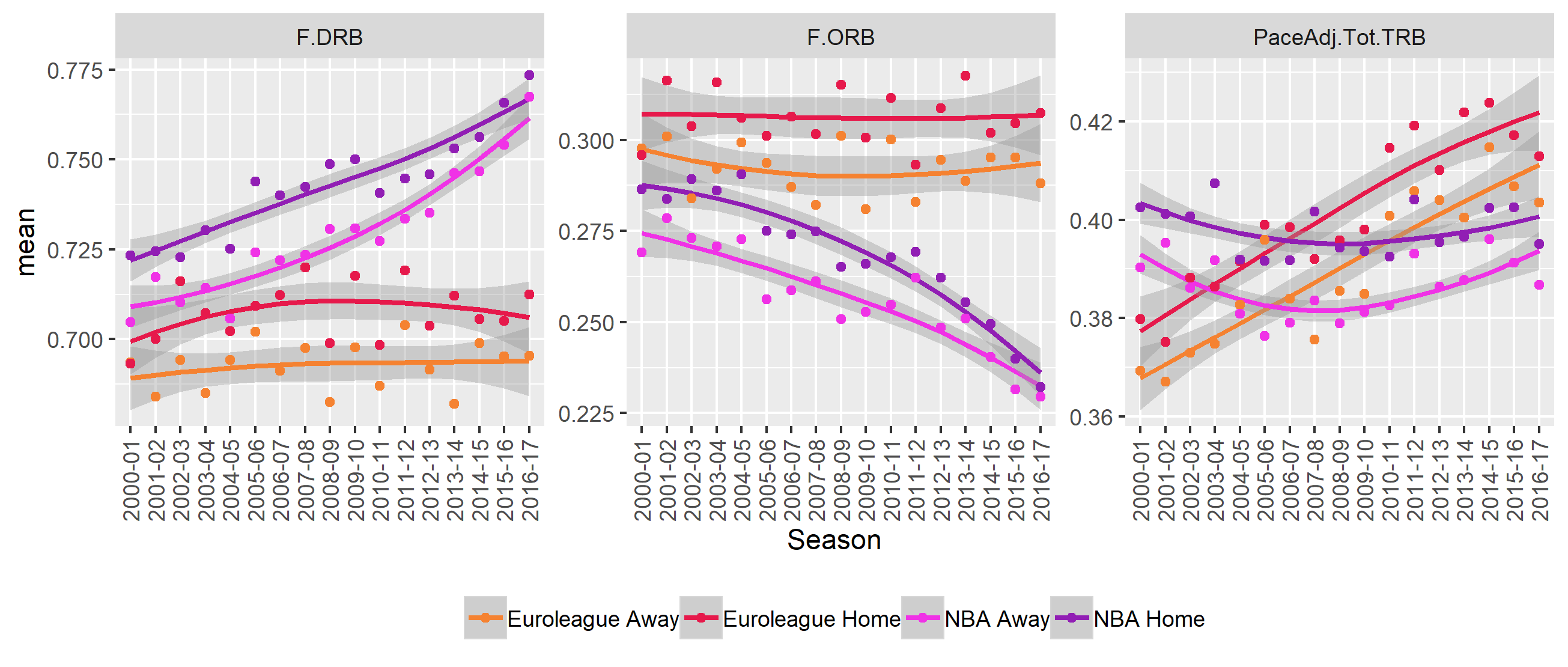

Supplement: S1 Figs — (ZIP) [file pone.0223524.s002.zip › Fig5_home_team.tiff]
