# Supplementary material for: Trends in NBA and Euroleague basketball: Analysis and comparison of statistical data from 2000 to 2017
Source: PLoS One. 2019 Oct 7;14(10):e0223524. doi: 10.1371/journal.pone.0223524 (PMC6779240; doi:10.1371/journal.pone.0223524)
Supplement: S1 Tables — (ZIP) [file pone.0223524.s003.zip › S3_Tables.pdf]

The first two columns are the estimated means (μ), followed by estimated difference in means (Δ_μ), and posterior probability that the difference is positive ($P_{\Delta_\mu > 0}$). The fifth and sixth column are the estimates slopes (s), followed by estimated difference in slope (Δ_s), and posterior probability that the difference is positive ($P_{\Delta_s > 0}$). Posterior probabilities under 0.01 or over 0.99 are marked with $*$. The second line for each variable contains the standard errors for the estimates. Note that the posterior distributions of μ and s are approximately normal so $\pm$ 2 standard errors is approximately the 95% Bayesian posterior confidence interval. Note that all estimates except for F.NP and Entropy.MIN are multiplied by 100 to simplify interpretation.

Euroleague vs NBA (all games)

	μ_{Euro}	μ_{NBA}	Δ_μ	$P_{\Delta_s>0}$	s_{Euro}	s_{NBA}	Δ_s	$P_{\Delta_s>0}$
F.NP	83.00	107.41	-24.41	*0.000	-0.15	0.16	-0.31	*0.001
	0.36	0.26	0.44		0.07	0.05	0.09	
Entropy.MIN	3.15	3.15	-0.01	0.175	0.01	0.01	0.01	*1.000
	0.00	0.01	0.01		0.00	0.00	0.00	
F.P3pct	35.59	35.16	0.43	0.924	-0.00	0.03	-0.04	0.263
	0.26	0.15	0.30		0.05	0.03	0.06	
F.P2pct	51.84	48.12	3.72	*1.000	-0.06	0.22	-0.28	*0.000
	0.19	0.18	0.26		0.04	0.04	0.05	
F.FTpct	73.33	75.61	-2.28	*0.000	0.17	0.06	0.11	0.967
	0.23	0.18	0.29		0.05	0.04	0.06	
PaceAdj.Tot.FTA	24.72	22.61	2.11	*1.000	-0.50	-0.17	-0.32	*0.000
	0.19	0.26	0.32		0.04	0.05	0.06	
PaceAdj.Tot.P2A	46.90	58.67	-11.76	*0.000	-0.01	-0.46	0.45	*1.000
	0.35	0.26	0.44		0.07	0.05	0.09	
PaceAdj.Tot.P3A	24.55	17.17	7.38	*1.000	0.32	0.60	-0.28	*0.000
	0.26	0.28	0.38		0.05	0.06	0.08	
PaceAdj.Tot.STL	9.77	7.03	2.75	*1.000	-0.35	-0.01	-0.34	*0.000
	0.34	0.07	0.34		0.07	0.01	0.07	
PaceAdj.Tot.AST	16.79	20.05	-3.26	*0.000	0.47	0.02	0.45	*1.000
	0.23	0.09	0.25		0.05	0.02	0.05	
PaceAdj.Tot.BLK	3.12	4.58	-1.47	*0.000	0.04	-0.02	0.06	*1.000
	0.03	0.05	0.06		0.01	0.01	0.01	
PaceAdj.Tot.TOV	16.17	12.85	3.31	*1.000	-0.06	-0.05	-0.00	0.469
	0.18	0.08	0.19		0.04	0.02	0.04	
F.FTperF	92.79	113.81	-21.01	*0.000	-1.14	0.13	-1.27	*0.000
	0.56	0.66	0.86		0.11	0.13	0.17	
PaceAdj.Tot.FCM	26.38	19.75	6.63	*1.000	-0.19	-0.17	-0.02	0.323
	0.14	0.15	0.21		0.03	0.03	0.04	
F.DRB	70.00	73.52	-3.52	*0.000	0.03	0.30	-0.27	*0.000
	0.16	0.16	0.22		0.03	0.03	0.05	
F.ORB	30.01	26.48	3.53	*1.000	-0.03	-0.30	0.27	*1.000
	0.14	0.16	0.22		0.03	0.03	0.04	
PaceAdj.Tot.TRB	39.54	39.22	0.33	0.945	0.28	0.01	0.27	*1.000
	0.16	0.13	0.21		0.03	0.03	0.04	

NBA regular season vs NBA playoffs

	μ_{Reg}	μ_{Pla}	Δ_μ	$P_{\Delta_s>0}$	s_{Reg}	s_{Pla}	Δ_s	$P_{\Delta_s>0}$
F.NP	107.57	105.15	2.41	*1.000	0.17	0.10	0.07	0.708
	0.25	0.55	0.60		0.05	0.11	0.12	
Entropy.MIN	3.16	3.08	0.08	*1.000	0.01	0.01	-0.00	0.110
	0.01	0.01	0.01		0.00	0.00	0.00	
F.P3pct	35.20	34.50	0.70	0.985	0.03	0.05	-0.01	0.418
	0.14	0.29	0.32		0.03	0.06	0.06	
F.P2pct	48.16	47.53	0.64	0.952	0.22	0.25	-0.03	0.354
	0.18	0.34	0.39		0.04	0.07	0.08	
F.FTpct	75.63	75.32	0.31	0.792	0.06	0.08	-0.02	0.413
	0.17	0.34	0.38		0.04	0.07	0.08	
PaceAdj.Tot.FTA	22.49	24.31	-1.82	*0.000	-0.17	-0.20	0.03	0.641
	0.26	0.28	0.38		0.05	0.06	0.08	
PaceAdj.Tot.P2A	58.78	56.96	1.82	*1.000	-0.46	-0.46	-0.01	0.473
	0.26	0.39	0.47		0.05	0.08	0.09	
PaceAdj.Tot.P3A	17.08	18.55	-1.47	*0.002	0.60	0.62	-0.02	0.430
	0.26	0.42	0.49		0.05	0.08	0.10	
PaceAdj.Tot.STL	7.04	6.77	0.27	0.987	-0.01	0.00	-0.01	0.255
	0.07	0.09	0.11		0.01	0.02	0.02	
PaceAdj.Tot.AST	20.14	18.73	1.41	*1.000	0.02	0.04	-0.02	0.299
	0.09	0.19	0.21		0.02	0.04	0.04	
PaceAdj.Tot.BLK	4.57	4.74	-0.17	0.072	-0.02	-0.02	-0.00	0.494
	0.05	0.10	0.11		0.01	0.02	0.02	
PaceAdj.Tot.TOV	12.89	12.32	0.57	*1.000	-0.05	-0.06	0.01	0.659
	0.08	0.12	0.15		0.02	0.03	0.03	
F.FTperF	113.89	112.54	1.35	0.902	0.14	0.07	0.07	0.623
	0.64	0.81	1.04		0.13	0.17	0.21	
PaceAdj.Tot.FCM	19.63	21.44	-1.80	*0.000	-0.17	-0.18	0.01	0.562
	0.16	0.19	0.25		0.03	0.04	0.05	
F.DRB	73.49	74.00	-0.52	0.031	0.30	0.28	0.02	0.633
	0.17	0.22	0.28		0.03	0.04	0.06	
F.ORB	26.52	25.99	0.52	0.967	-0.30	-0.28	-0.02	0.357
	0.17	0.21	0.28		0.03	0.04	0.06	
PaceAdj.Tot.TRB	39.19	39.61	-0.42	0.044	0.01	-0.04	0.05	0.846
	0.13	0.21	0.25		0.03	0.04	0.05	

Euroleague regular season vs Euroleague play-offs

	μ_{Reg}	μ_{Pla}	Δ_μ	$P_{\Delta_s>0}$	s_{Reg}	s_{Pla}	Δ_s	$P_{\Delta_s>0}$
F.NP	83.38	82.22	1.16	0.981	-0.13	-0.14	0.02	0.552
	0.38	0.39	0.55		0.08	0.08	0.11	
Entropy.MIN	3.14	3.15	-0.01	0.162	0.01	0.01	0.00	0.622
	0.00	0.01	0.01		0.00	0.00	0.00	
F.P3pct	35.39	35.96	-0.57	0.082	-0.01	-0.05	0.04	0.699
	0.28	0.30	0.41		0.06	0.06	0.09	
F.P2pct	51.87	51.75	0.11	0.640	-0.06	-0.07	0.01	0.532
	0.20	0.24	0.31		0.04	0.05	0.06	
F.FTpct	73.21	73.46	-0.26	0.261	0.16	0.17	-0.01	0.433
	0.25	0.35	0.43		0.05	0.07	0.09	
PaceAdj.Tot.FTA	24.64	24.95	-0.31	0.215	-0.51	-0.48	-0.03	0.377
	0.21	0.34	0.40		0.04	0.07	0.08	
PaceAdj.Tot.P2A	47.02	46.51	0.51	0.834	-0.03	0.03	-0.07	0.269
	0.37	0.38	0.53		0.08	0.08	0.11	
PaceAdj.Tot.P3A	24.29	25.11	-0.82	0.023	0.31	0.28	0.03	0.646
	0.28	0.29	0.40		0.06	0.06	0.08	
PaceAdj.Tot.STL	9.78	9.87	-0.09	0.418	-0.32	-0.45	0.13	0.914
	0.34	0.28	0.44		0.07	0.06	0.09	
PaceAdj.Tot.AST	16.71	16.91	-0.20	0.276	0.46	0.46	-0.00	0.499
	0.25	0.24	0.35		0.05	0.05	0.07	
PaceAdj.Tot.BLK	3.07	3.28	-0.21	*0.000	0.04	0.05	-0.01	0.190
	0.03	0.02	0.04		0.01	0.01	0.01	
PaceAdj.Tot.TOV	16.36	15.87	0.49	0.972	-0.03	-0.09	0.06	0.884
	0.20	0.16	0.26		0.04	0.03	0.05	
F.FTperF	93.02	92.08	0.94	0.861	-1.17	-1.05	-0.12	0.258
	0.57	0.67	0.88		0.12	0.14	0.18	
PaceAdj.Tot.FCM	26.22	26.85	-0.63	0.021	-0.19	-0.19	-0.01	0.449
	0.17	0.25	0.31		0.04	0.05	0.07	
F.DRB	69.85	70.44	-0.59	0.019	0.05	-0.05	0.10	0.963
	0.19	0.21	0.28		0.04	0.04	0.06	
F.ORB	30.15	29.58	0.58	0.975	-0.06	0.05	-0.10	0.045
	0.19	0.23	0.30		0.04	0.05	0.06	
PaceAdj.Tot.TRB	39.30	40.17	-0.88	*0.001	0.29	0.23	0.07	0.916
	0.17	0.17	0.24		0.03	0.04	0.05	

NBA home team vs NBA away team

	μ_{Home}	μ_{Away}	Δ_μ	$P_{\Delta_s>0}$	s_{Home}	s_{Away}	Δ_s	$P_{\Delta_s>0}$
F.NP	107.57	107.25	0.32	0.808	0.15	0.17	-0.02	0.394
	0.26	0.28	0.38		0.05	0.06	0.08	
Entropy.MIN	3.15	3.16	-0.01	0.188	0.01	0.01	0.00	0.643
	0.01	0.01	0.01		0.00	0.00	0.00	
F.P3pct	35.57	34.74	0.83	*0.999	0.04	0.03	0.01	0.608
	0.16	0.20	0.25		0.03	0.04	0.05	
F.P2pct	48.81	47.44	1.36	*1.000	0.20	0.23	-0.03	0.288
	0.19	0.19	0.27		0.04	0.04	0.05	
F.FTpct	75.73	75.49	0.24	0.833	0.06	0.06	-0.00	0.496
	0.18	0.18	0.25		0.04	0.04	0.05	
PaceAdj.Tot.FTA	23.10	22.12	0.98	*0.995	-0.17	-0.18	0.02	0.581
	0.28	0.24	0.37		0.06	0.05	0.08	
PaceAdj.Tot.P2A	58.65	58.68	-0.03	0.473	-0.46	-0.46	-0.00	0.490
	0.26	0.26	0.37		0.05	0.05	0.07	
PaceAdj.Tot.P3A	17.13	17.22	-0.09	0.403	0.60	0.61	-0.01	0.424
	0.28	0.28	0.39		0.06	0.06	0.08	
PaceAdj.Tot.STL	7.07	6.99	0.08	0.817	-0.01	-0.01	-0.01	0.393
	0.07	0.07	0.10		0.01	0.01	0.02	
PaceAdj.Tot.AST	20.76	19.35	1.41	*1.000	-0.01	0.04	-0.04	0.048
	0.10	0.09	0.13		0.02	0.02	0.03	
PaceAdj.Tot.BLK	4.86	4.31	0.55	*1.000	-0.03	-0.01	-0.02	0.061
	0.05	0.05	0.07		0.01	0.01	0.01	
PaceAdj.Tot.TOV	12.66	13.05	-0.38	*0.001	-0.05	-0.06	0.01	0.657
	0.08	0.09	0.12		0.02	0.02	0.02	
F.FTperF	114.39	113.20	1.19	0.915	0.13	0.14	-0.01	0.478
	0.64	0.62	0.89		0.13	0.13	0.18	
PaceAdj.Tot.FCM	19.37	20.13	-0.76	*0.000	-0.18	-0.17	-0.01	0.409
	0.14	0.18	0.22		0.03	0.04	0.04	
F.DRB	74.14	72.90	1.25	*1.000	0.27	0.32	-0.05	0.127
	0.17	0.17	0.24		0.03	0.03	0.05	
F.ORB	27.11	25.86	1.25	*1.000	-0.32	-0.27	-0.05	0.125
	0.17	0.16	0.24		0.03	0.03	0.05	
PaceAdj.Tot.TRB	39.81	38.63	1.18	*1.000	-0.01	0.03	-0.03	0.184
	0.14	0.13	0.19		0.03	0.03	0.04	

Euroleague home team vs Euroleague away team

	μ_{Home}	μ_{Away}	Δ_μ	$P_{\Delta_s>0}$	s_{Home}	s_{Away}	Δ_s	$P_{\Delta_s>0}$
F.NP	83.22	82.75	0.47	0.826	-0.14	-0.15	0.02	0.565
	0.36	0.37	0.52		0.07	0.08	0.11	
Entropy.MIN	3.15	3.14	0.02	0.987	0.01	0.01	-0.00	0.327
	0.01	0.00	0.01		0.00	0.00	0.00	
F.P3pct	36.16	35.05	1.11	*0.996	-0.02	0.01	-0.03	0.339
	0.22	0.32	0.39		0.05	0.06	0.08	
F.P2pct	52.78	50.88	1.90	*1.000	-0.06	-0.06	-0.01	0.455
	0.22	0.20	0.29		0.04	0.04	0.06	
F.FTpct	73.74	72.91	0.83	0.987	0.18	0.16	0.02	0.603
	0.25	0.29	0.38		0.05	0.06	0.08	
PaceAdj.Tot.FTA	25.34	24.09	1.25	*1.000	-0.45	-0.54	0.08	0.922
	0.21	0.22	0.30		0.04	0.04	0.06	
PaceAdj.Tot.P2A	47.03	46.78	0.25	0.697	-0.02	-0.01	-0.01	0.451
	0.32	0.40	0.51		0.07	0.08	0.10	
PaceAdj.Tot.P3A	24.71	24.40	0.31	0.801	0.29	0.35	-0.06	0.197
	0.26	0.28	0.38		0.05	0.06	0.08	
PaceAdj.Tot.STL	10.19	9.35	0.84	0.962	-0.39	-0.31	-0.08	0.180
	0.34	0.32	0.47		0.07	0.07	0.10	
PaceAdj.Tot.AST	17.79	15.81	1.97	*1.000	0.42	0.51	-0.08	0.103
	0.26	0.22	0.34		0.05	0.04	0.07	
PaceAdj.Tot.BLK	3.38	2.86	0.52	*1.000	0.04	0.05	-0.01	0.178
	0.05	0.04	0.07		0.01	0.01	0.01	
PaceAdj.Tot.TOV	15.56	16.76	-1.20	*0.000	-0.04	-0.07	0.04	0.756
	0.16	0.20	0.26		0.03	0.04	0.05	
F.FTperF	94.49	91.02	3.47	*1.000	-1.04	-1.22	0.18	0.865
	0.61	0.58	0.83		0.12	0.12	0.17	
PaceAdj.Tot.FCM	26.01	26.75	-0.74	*0.001	-0.22	-0.17	-0.05	0.098
	0.16	0.14	0.21		0.03	0.03	0.04	
F.DRB	70.74	69.25	1.50	*1.000	0.02	0.04	-0.02	0.371
	0.19	0.19	0.27		0.04	0.04	0.05	
F.ORB	30.76	29.27	1.49	*1.000	-0.04	-0.02	-0.02	0.377
	0.19	0.18	0.26		0.04	0.04	0.05	
PaceAdj.Tot.TRB	40.13	38.95	1.18	*1.000	0.28	0.29	-0.01	0.453
	0.18	0.18	0.25		0.04	0.04	0.05	
